# Supplementary material for: Increased Frataxin Expression Induced in Friedreich Ataxia Cells by Platinum TALE-VP64s or Platinum TALE-SunTag
Source: Mol Ther Nucleic Acids. 2018 Apr 27;12:19–32. doi: 10.1016/j.omtn.2018.04.009 (PMC6019861; doi:10.1016/j.omtn.2018.04.009)
Supplement: Document S1. Supplemental Results, Figures S1–S8, and Table S1 [file mmc1.pdf]

OMTN, Volume *12*

Supplemental Information

Increased Frataxin Expression Induced in Friedreich Ataxia Cells by Platinum TALE-VP64s or Platinum TALE-SunTag

Khadija Cherif, Catherine Gérard, Joël Rousseau, Dominique L. Ouellet, Pierre Chapdelaine, and Jacques P. Tremblay

Increased frataxin expression induced in Friedreich ataxia cells

by platinum TALE-VP64s or platinum TALE-SunTag

Khadija Cherif, Catherine Gérard, Joël Rousseau, Dominique L. Ouellet,

Pierre Chapdelaine and Jacques P. Tremblay*

Centre de Recherche du CHU de Québec-Université Laval and Département

de Médecine Moléculaire de l'Université Laval Québec, Canada.

Supplementary results

Assembly of the various TALEN

The assembled TALENs were verified by PCR (Figure 1S).

Lack of improvement in the specificity and affinity of plTALEs with new nRVD sequences (nplTALEs)

To further improve our therapeutic approach, we constructed plTALEs with new RVDs (nRVDs) [1] (i.e., nplTALE$_{VP64s}$) to increase the specificity and affinity of RVDs. First, the mutations of the nucleotides encoding the amino acid at position 12 coding for the RVD were made in the plasmids pCR3.1-plTALE$_{VP64-8-15}$ and pCR3.1-plTALE-$_{VP64-F4-15}$. These mutations replaced the RVDs NI, NG, and NN by CI, HG and EN respectively to generate the new RVDs. These new RVDs also contained 34 amino acids and were different from normal RVDs only by substitutions of the nucleotides encoding the amino acid in position 12. These base changes were AAC to TGC (to modify the asparagine into a cysteine, thus changing the RVDs NI into CI), AAC to CAC (to modify the asparagine into a histidine, thus changing the RVD NG into HG) and AAC to GAG (to modify the asparagine into a glutamic acid, thus changing the RVD NN into EN) (Figure S2A).

Thereafter, 2 new nplTALE$_{VP64s}$ with 15 nRVDs were built according to the same protocol that was used to build the plTALE$_{VP64s}$. These nplTALE$_{VP64s}$ targeted 2 sequences of 15 nucleotides sequences (i.e., *FXN* sequences 8 and F4) (Figure S2B). These 2 nplTALE$_{VP64s}$ were nucleofected in FRDA4078 cells to analyze 3 days later their effects on the transcriptional activity of the *FXN* gene. QRT-PCR analyzes (or analysis) were performed to quantify the number of *FXN* mRNA

copies. The transcriptional activity was normalized to GAPDH and HPRT, and compared to negative controls, i.e., FRDA4078 cells untreated or treated with an empty pCR3.1 plasmid (Figure S2C). The results showed that *FXN* mRNAs was not increased in the cells treated with $nplTALE_{VP64\text{-}8\text{-}15}$ and $nplTALE_{VP64\text{-}F4\text{-}15}$ compared to the negative controls. The *FXN* mRNA normalized with GAPDH and HPRT mRNAs did not change relative to the negative controls. Thus these 2 $nplTALE_{VP64s}$ with nRVDs (i.e., CI, NN and NG) did not activate the *FXN* gene in FRDA4078 cells. The efficacy of these 2 $nplTALE_{VP64s}$ was decreased compared to $plTALEs_{VP64s}$ targeting the same sequences.

Epigenetic change of the *FXN* gene with p300 acetyltransferase fused with plTALEs

P300 is a histone acetyltransferase (HAT) that regulates the transcription and remodeling of chromatin by histone acetylation. We fused p300 with plTALEs targeting either the transcription initiation region or a sequence in intron 1 of the *FXN* gene. These $plTALE_{p300}$ have been reported to be more effective to induce gene transcription after histone H3-lysine acetylation [2]. We constructed 3 $plTALE_{p300s}$ (Figure S2D) targeting 3 sequences (i.e., 6, 8 and F4) of the *FXN* gene. FRDA4087 cells were nucleofected with each of these 3 $plTALE_{p300s}$. The *FXN* gene transcription of these treated cells was compared 3 days later with the negative controls (untreated cells and cells treated with empty pCR3.1) (Figure S2E). When the *FXN* mRNA was normalized with either GAPDH or HPRT, the increases obtained with $plTALE_{p300\text{-}6\text{-}15}$ and with $plTALE_{p300\text{-}8\text{-}15}$ reached significant levels, but their effects were not as much as the effect of $plTALE_{VP64.}$

The effects of $plTALE_{p300s}$ were very low compared to those of $plTALE_{VP64s}$ targeting the same sequences. Thus, to activate the endogenous *FXN* gene, VP64 effectors are more potent than p300 effectors fused with plTALEs targeting sequences either in the promoter or in intron 1 of the *FXN* gene.

Study of the synergistic effects of plTALE$_{VP64s}$ and plTALE$_{p300}$ on *FXN* gene transcription in FRDA4078 cells

To study the synergistic effect of several plTALE$_{VP64}$ together, we treated FRDA4078 with the combinations of the 2, 3 or 4 most effective plTALE$_{VP64s}$. The *FXN* mRNAs increased even more when we treated these cells with several plTALE$_{VP64s}$ compared to the negative controls. The synergistic effect increased the *FXN* transcription normalized with GAPDH up to 3 folds when 4 effectors targeting sequences 6, 8, 10 and F4 were used (Figure 2A and B).

Analysis of the expression of the frataxin protein in cells treated by the different combinations of plTALE$_{VP64s}$ and in the negative control showed an increase of slightly more than 2.5 folds with the combination of plTALE$_{6-15}$ and plTALE$_{8-15}$. The combination of 3 plTALEs only increased frataxin expression by 1.9 fold over the negative control (Figure 2D and E). Therefore, these 3 plTALEs do not have a synergistic effect on the expression of frataxin compared with the effect of a single plTALE (Figure 2D and E).

The synergistic effects of the various combinations of plTALE$_{VP64s}$ with plTALE$_{p300s}$ and several plTALE$_{p300s}$ together, targeting the different sequences of the *FXN* gene, were investigated in FRDA4078 cells (Figure S3A and B). The transcriptional activity of the *FXN* gene was increased about 2 folds by two combinations: plTALE$_{VP64-6-15}$ with plTALE$_{p300-8-15}$ and plTALE$_{VP64-8-15}$ with plTALE$_{p300-6-15}$. Our previous results had shown that plTALE$_{VP64-6-15}$ or plTALE$_{VP64-8-15}$ alone also increased the transcriptional activity of the *FXN* gene in the FRDA4078 cells by only 2 folds. Thus, the effect of the combination of these 2 plTALEs (VP64 + p300) did not activate the *FXN* transcription more than a plTALE$_{VP64}$ alone. However, the combination of plTALE$_{p300-6-15}$ with plTALE$_{p300-8-15}$ significantly increased *FXN* transcription by 2 folds compared to the controls showing a small synergistic effect. This activity was equivalent to the effect of a single plTALE$_{VP64}$ with 15 RVD targeting sequences 6, 8 or F4.

Labelling the target locus of gene frataxin with plTALE$_{ST10X}$ expressed with the CAG or CMV promoter

We subsequently constructed pAAV-plTALE$_{ST10X-6-15}$ and pAAV-plTALE$_{ST10X-8-15}$. We also made the pAAV-scFv (pAAV-scFv-sfGFP-VP64-GB1-NLS). These pAAV plasmids contained a CAG promoter while our previous pCR3.1 plasmid contained a CMV promoter. The new constructions were initially tested *in vitro* in FRDA4078 cells using the same nucleofection protocol to compare their effectiveness relative to the previous pCR3.1 plasmids. The plTALE$_{ST10X}$ effectors expressed by pAAV and pCR3.1 similarly increased *FXN* transcriptional activity (Figure S4A and B). *FXN* transcription was increased by more than 8 folds by plTALE$_{ST10X-6-15}$ expressed either by pCR3.1 or by pAAV. This activity was also increased about 5 folds by plTALE$_{ST10X-8-15}$ expressed by pAAV and by about 8 folds by plTALE$_{ST10X-8-15}$ expressed by pCR3.1. These two effectors expressed either by pCR3.1 or pAAV synergistically increased the *FXN* transcription more than 18 folds.

Microscopic observation of the nuclei of the cells treated with this ST system revealed a much brighter sfGFP fluorescence in the nuclei of the cells nucleofected with a combination plTALE$_{ST10X-6-15}$ and plTALE$_{ST10X-8-15}$, which recruited a total of 20 scFv-sfGFP-VP64s (i.e., 10 tags for each plTALE) compared with the negative controls treated with a plasmid, which did not express the plTALE$_{ST10X}$, and a plasmid, which expressed only scFv-sfGFP-VP64 (Figure S4C). The sfGFP of the latter was expressed in the cytoplasm and in the nucleus but did not labelled the nuclei intensively in the absence of plTALE$_{ST}$. With the SunTag system, the two plTALE$_{ST10X}$ (targeting sequences 6 and 8 separated by 10 nucleotides) recruited of 20 scFv-sfGFP-VP64 leading to an intense fluorescent labeling of the nuclei confirming the *FXN* targeting by the 2 plTALE$_{ST10X}$.

In vitro treatment of FRDA4078 cells with pAAV-plTALE$_{ST10Xs}$ or pCR3.1-plTALE$_{ST10Xs}$ activated the expression of the endogenous *FXN* gene. However, microscopic observations of the cells treated with these effectors showed a much brighter nuclear labeling of cells treated with

pCR3.1 plasmids than with pAAV plasmids (Figure S4D). The signal difference may be due to the different promoters.

Bibliography

1. Miller, JC, Zhang, L, Xia, DF, Campo, JJ, Ankoudinova, IV, Guschin, DY, *et al.* (2015). Improved specificity of TALE-based genome editing using an expanded RVD repertoire. *Nature Methods* **12**: 465.
2. Hilton, IB, D'Ippolito, AM, Vockley, CM, Thakore, PI, Crawford, GE, Reddy, TE, *et al.* (2015). Epigenome editing by a CRISPR/Cas9-based acetyltransferase activates genes from promoters and enhancers. *Nature biotechnology* **33**: 510-517.

Figures and legends

Figure S1

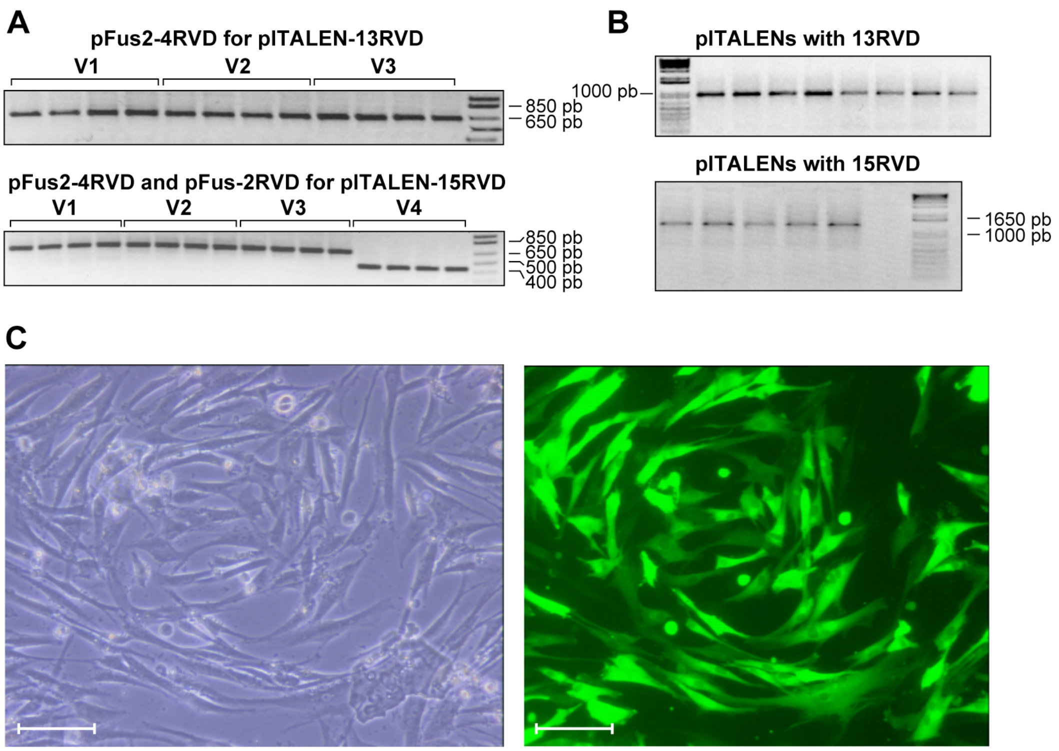

Figure S1: Validation of the construction of plTALE$_{VP64s}$ targeting different sequences of the *FXN* gene and nucleofected in FRDA4078 fibroblasts.

A) The figure illustrates the PCR amplification of sequences containing 4 RVDs (V1, V2 and V3) corresponding to the first 12 RVDs of plTALEN-13RVD or plTALEN-15RVD. The V4 is the PCR amplification of the assembly of 2 RVDs corresponding to the thirteenth and fourteenth RVDs of plTALEN-15RVD. **B)** PCR to select the adequate final assembly of plTALENs with 13 RVDs (1332 pb) and 15 RVDs (1532 pb). **C)** Representative image of FRDA4078 fibroblasts (left side in phase contrast and right side in fluorescence), 24 h after nucleofection with a plasmid containing the GFP gene. Scale bars in C are 100 μm.

Figure S2

A

Target Nucleotide	Normal RVD	New RVD
A	**NI**<br>**AA**C ATA	**CI**<br>**TG**C ATA
T	**NG**<br>**A**AC GGC	**HG**<br>**C**AC GGC
C	**HD**<br>CAC GAT	**HD**<br>CAC GAT
G	**NN**<br>**AAC** AAC	**EN**<br>**GAG** AAC

B

npITALE-VP64	Target sequence	RVD sequence
**npITALE-VP64-8-15**	TCCTGAGGTCTAACCT	HD HD HG EN CI EN EN HG HD HG CI CI HD HD HG
**npITALE-VP64-F4-15**	TGCGCAGGGAGGCGCC	EN HD EN HD CI EN EN EN CI EN EN HD EN HD HD

C

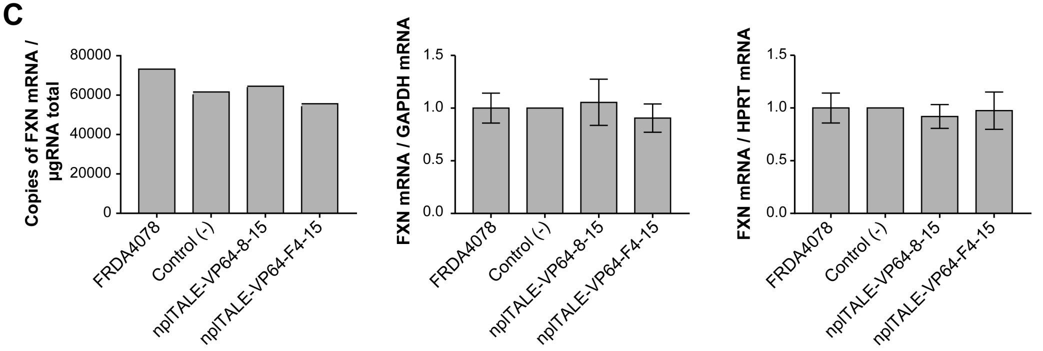

D

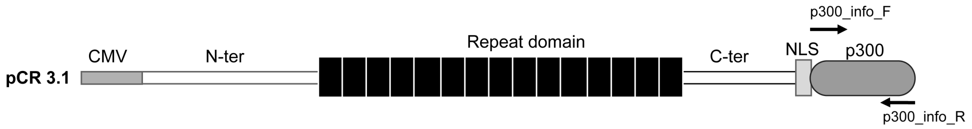

E

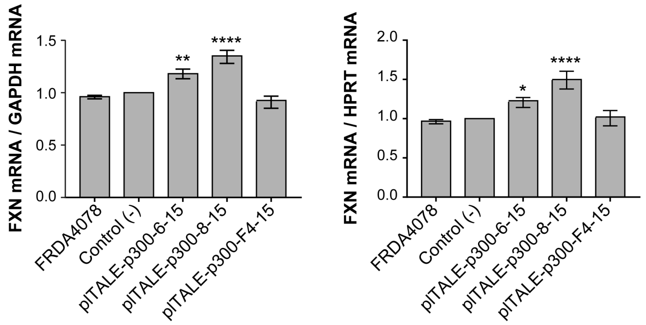

Figure S2: Study of the effects of the npITALE$_{VP64s}$ and pITALE$_{p300s}$.

A) The amino acid pairs and the codons of the normal and new RVDs targeting a specific nucleotide. **B)** List of npITALE$_{VP64s}$ with their new RVD sequences and their target sequences. **C)** *FXN* gene mRNA in control cells and in FRDA4078 cells treated with npITALE$_{VP64}$. **D)** Scheme

of plTALE$_{P300}$ in pCR3.1 containing the CMV promoter, N-ter, repeat domain, C-ter, NLS and p300. **E)** *FXN* gene transcriptional activity in control cells and in cells treated with a plTALE$_{p300}$. This activity was demonstrated by comparing in control cells and in the treated cells the number of copies of *FXN* mRNA, the number *FXN* mRNAs normalized with GAPDH mRNAs or with the HPRT mRNA. Statistics: $p < 0.05$*, $p < 0.003$** and $p < 0.0001$****.

Figure S3

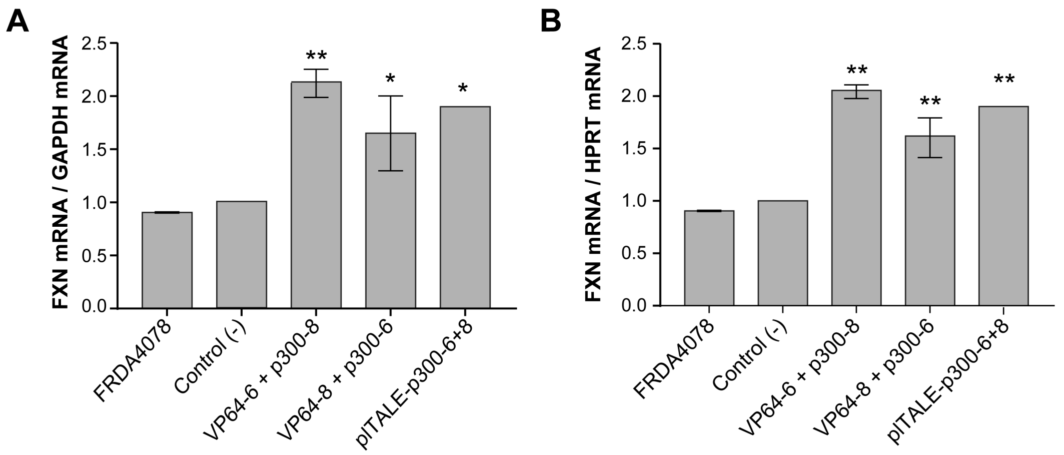

Figure S3 Absence of synergistic effects of plTALE$_{VP64}$ and plTALE$_{p300}$ in FRDA4078 fibroblasts.

A) and **B)** *FXN* gene transcriptional activity with the combined effects of plTALE$_{VP64}$ + plTALE$_{p300}$ or combined effects of 2 plTALE$_{p300s}$, which targeted sequences 6 and 8. The effectors used for this test were plTALE$_{VP64-6-15}$, plTALE$_{VP64-8-15}$, plTALE$_{p300-6-15}$ and plTALE$_{p300-8-15}$. Statistics: $p < 0.05$* and $p < 0.003$**.

Figure S4

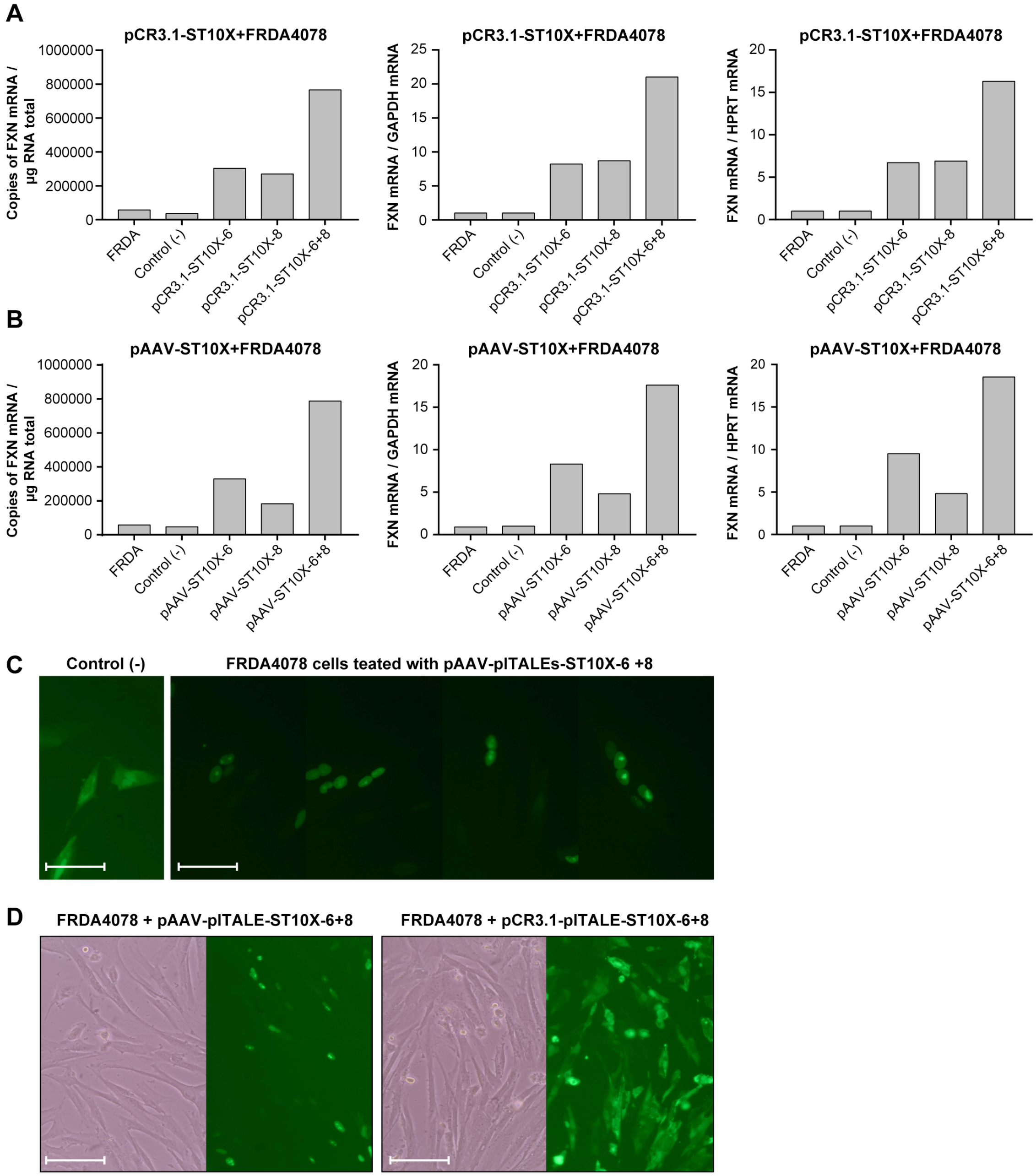

Figure S4: Induction of *FXN* gene in FRDA4078 cells with plTALE$_{STs}$ expressed by CMV or CAG promoter, and their nucleus location.

A) and **B)** *FXN* gene mRNA normalized with GAPDH or HPRT mRNA in untreated FRDA4078 (control -) or treated with 1 or 2 plTALE$_{ST10Xs}$ and scFV-sfGFP-VP64-GB1-NLS (scFv)

expressed by the pCR3.1 plasmid (promoter CMV) or the pAAV plasmid (promoter CAG). **C)** Left side: GFP fluorescence in a negative control, i.e., FRDA4078 cells treated with the pAAV-scFV-GFP plasmid, which did not express by the plTALE and with pAAV-scFv. In these cells, the GFP green fluorescence is uniform over the whole cells there no intense labelling of the nuclei. Right side: FRDA4078 cells treated with pAAV-plTALE$_{ST10X-6}$ + pAAV-plTALE$_{ST10X-8}$ + pAAV-scFv. These cells contain brighter nuclei due to plTALE$_{ST10Xs}$ targeting the specific nucleotide sequences 6 and 8 and each ST recruiting 10 scFv-sfGFP-VP64-GB1 complexes (for a total of 20 complexes). **D)** Phase contrast and GFP fluorescence of FRDA4078 cells treated with different plTALE$_{ST10X}$. Left side: FRDA4078 cells treated with pAAV-plTALE$_{ST10X-6-15}$ + pAAV-plTALE$_{ST10X-8-15}$ + pAAV-scFv, the GFP signal is localized at the plTALE targeted loci that fix 10 GFP-FV64 for each plTALE (a total of 20 GFP-VP64) leading to intense fluorescence of the nuclei. Right side: FRDA4078 cells treated with pCR3.1-plTALE$_{ST10X-6-15}$ + pCR3.1-plTALE$_{ST10X-8-15}$ + pCR3.1-scFV led to recruitment of GFP and to intense labelling of the nuclei. Scale bars in C are 50 μm and in D 100 μm.

Figure S5

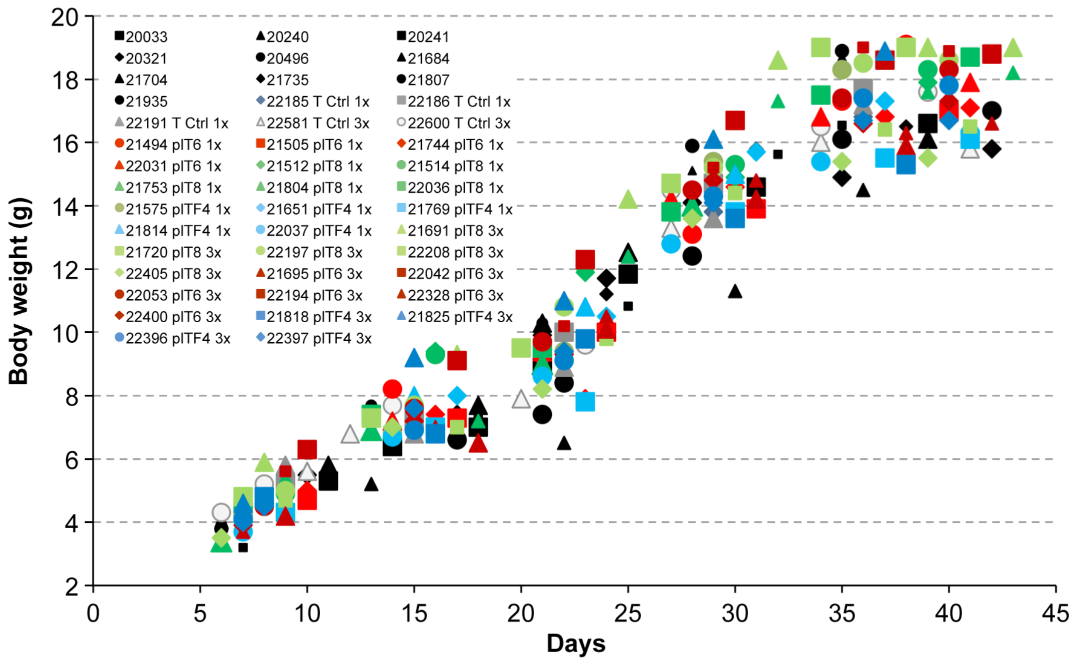

Figure S5: Body weight of untreated mice and mice treated with the different viruses.

Representative diagram of the weights of different YG8R mice treated with AAV9 that express plTALE$_{VP64\text{-}6\text{-}15}$, plTALE$_{VP64\text{-}8\text{-}15}$ or plTALE$_{VP64\text{-}F4}$ compared to control YG8R mice either untreated or treated with AAV9 (AAV9-ctrl), which did not express plTALE$_{VP64}$.

Figure S6

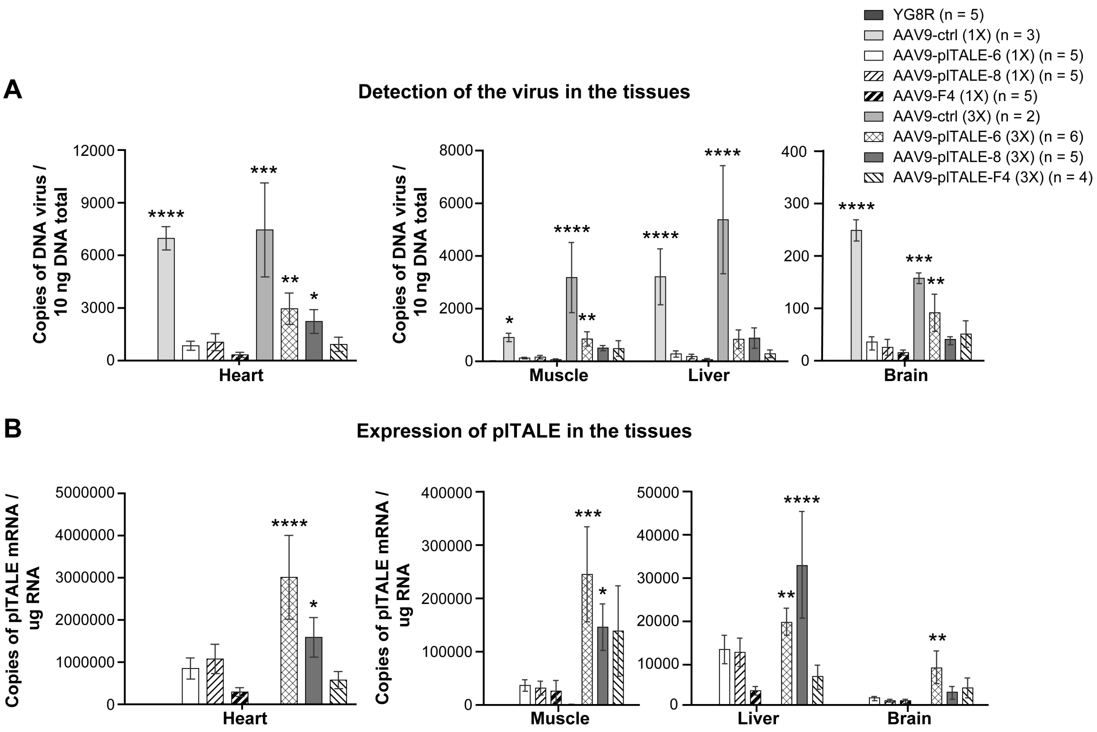

Figure S6: The distribution and expression of AAV-plTALE$_{VP64}$ *in vivo* in YG8R mice

A) Quantification of AAV9 genome in tissues: muscle, heart, liver and brain compared to untreated mice (YG8R). **B)** The expression level of plTALE$_{VP64}$ in tissues: muscle, heart, liver and brain compared to untreated mice (YG8R). Statistics: $p < 0.05$*, $p < 0.003$**, $p < 0.0003$*** and $p < 0.0001$****.

Figure S7

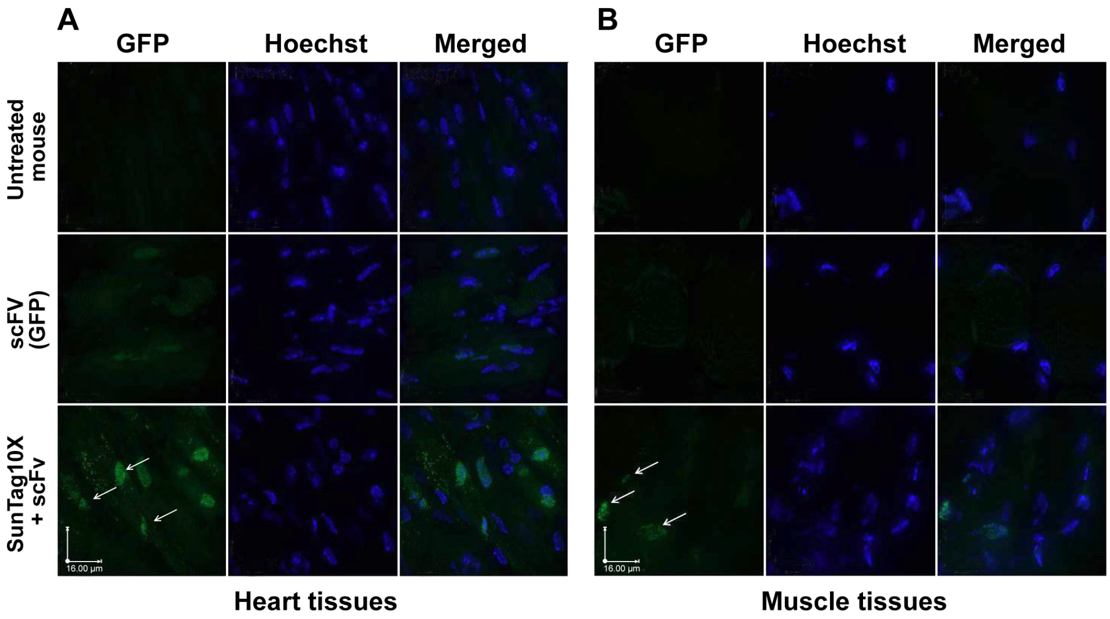

Figure S7: Labeling of the nuclei in mice treated with plTALE$_{ST10X}$.

A) and **B**) YG8R mice were injected IP with 3 different AAV viruses (i.e., 3 X 10^{11} v.p. AAV9-plTALE$_{ST10X-6}$ + 3 X 10^{11} v.p. AAV9-plTALE$_{ST10X-8}$ + 3 X 10^{11} v.p. AAV9-scFV-sfGFP-VP64). The confocal microscopy images illustrate the localization of the 20 scFV-sfGFP-VP64s recruited by plTALE$_{ST10X-6}$ and plTALE$_{ST10X-8}$ targeting the *FXN* gene promoter. The GFP fluorescence is concentrated at the nuclei (arrows) only in the heart (**A**) and the muscles (**B**) treated with AAV9s that expressed both plTALE$_{ST10X}$ and scFV-sfGFP-VP64 compared to control mouse cells where scFV-sfGFP-VP64 is expressed throughout the cytoplasm and the nucleus of the cells in the absence of plTALE$_{ST10X}$s. (Scale bar: 16 µm).

Figure S8

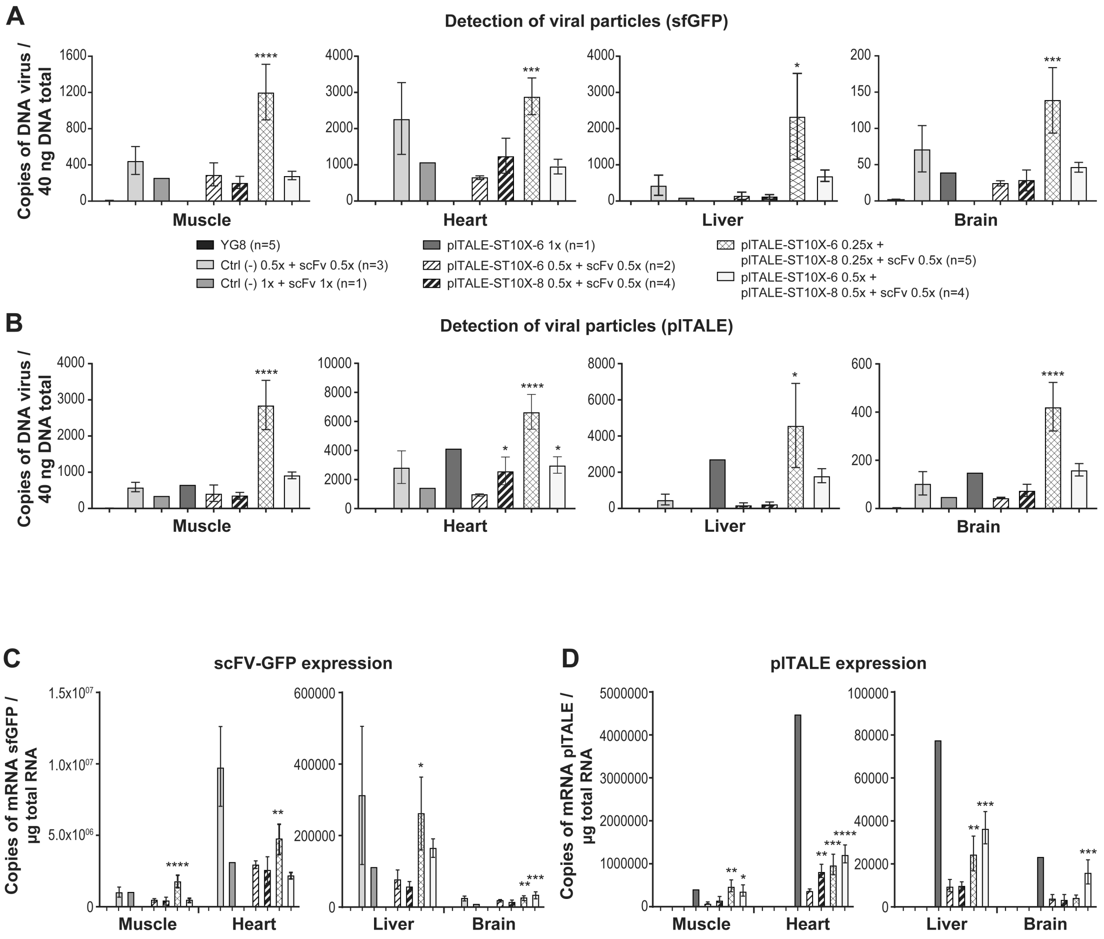

Figure S8: The distribution and expression of AAV-plTALE$_{ST10X}$ *in vivo* in YG8R mice

A) and **B)** Number of copies of AAV9 viruses, expressing either scFV or a plTALE$_{ST10X}$, in various organs of control and treated mice. **C)** and **D)** The number copies of plTALE$_{ST10X}$ mRNA and scFV-GFP mRNA in various organs of mice that received different treatments and untreated mice. Statistics: $p < 0.05$*, $p < 0.003$**, $p < 0.0003$*** and $p < 0.0001$****.

Supplementary Table 1

Gene Symbol	Description	GenBank	Size (pb)	Primer sequence 5' 3' SIAS
FXN	Homo sapiens frataxin (FXN) , nuclear gene encoding mitochondrial prote in, common region for the 3 transcrpits	NM 000144	106	AAGCCATACACGTTTGAGGACTA/TTGGCGTCTGCTTGTTGATCA
TALES			136	GAGTCGGTGTCACAGAACTCGAG/CGCCTGGTCAGGTGTCTGAGTTG
GFP			157	CCTCGTGACCACCCTGACCTAC/CTCGGCGCGGGTCTTGTAGTT
sfGFP			153	ACGCGTGCTGAAGTCAAGTTTG/CTTTTGTTTGTCTGCCGTGATGTATAC
HPRT1	Mus musculus hypoxanthine guanine phosphoribosyl transferase 1 (Hprt1)	NM 013556	106	CAGGACTGAAAGACTTGCTCGAGAT/CAGCAGGTCAGCAAAGAACTTATAGC
GAPDH	Mus musculus glyceralde-hyde -3-phosphate dehy-drogenase	NM_008084	194	GGCTGCCCAGAACATCATCCCT/ATGCCTGCTTCACCACCTTCTTG
ADNg		NT_039239	209	CACCCCTTAAGAGACCCATGTT/CCCTGCAGAGACCTTAGAAAAC
