# Supplementary material for: Increased Frataxin Expression Induced in Friedreich Ataxia Cells by Platinum TALE-VP64s or Platinum TALE-SunTag
Source: Mol Ther Nucleic Acids. 2018 Apr 27;12:19–32. doi: 10.1016/j.omtn.2018.04.009 (PMC6019861; doi:10.1016/j.omtn.2018.04.009)
Supplement: Document S2. Article plus Supplemental Information [file mmc2.pdf]

Molecular Therapy
Nucleic Acids
Original Article

Increased Frataxin Expression Induced in Friedreich Ataxia Cells by Platinum TALE-VP64s or Platinum TALE-SunTag

Khadija Cherif,[1,2] Catherine Gérard,[1,2] Joël Rousseau,[1,2] Dominique L. Ouellet,[1,2] Pierre Chapdelaine,[1,2] and Jacques P. Tremblay[1,2]

[1]Centre de Recherche du CHU, Québec-Université Laval, Québec, QC, Canada; [2]Département de Médecine Moléculaire, l'Université Laval Québec, Québec, QC, Canada

Frataxin gene (*FXN*) expression is reduced in Friedreich's ataxia patients due to an increase in the number of GAA trinucleotides in intron 1. The frataxin protein, encoded by that gene, plays an important role in mitochondria's iron metabolism. Platinum TALE (plTALE) proteins targeting the regulatory region of the *FXN* gene, fused with a transcriptional activator (TA) such as VP64 or P300, were used to increase the expression of that gene. Many effectors, $plTALE_{VP64}$, $plTALE_{p300}$, and $plTALE_{SunTag}$, targeting 14 sequences of the *FXN* gene promoter or intron 1 were produced. This permitted selection of 3 $plTALE_{VP64s}$ and 2 $plTALE_{SunTag}$ that increased *FXN* gene expression by up to 19-fold in different Friedreich ataxia (FRDA) primary fibroblasts. Adeno-associated viruses were used to deliver the best effectors to the YG8R mouse model to validate their efficiencies *in vivo*. Our results showed that these selected $plTALE_{VP64s}$ or $plTALE_{SunTag}$ induced transcriptional activity of the endogenous *FXN* gene as well as expression of the frataxin protein in YG8R mouse heart by 10-fold and in skeletal muscles by up to 35-fold. The aconitase activity was positively modulated by the frataxin level in mitochondria, and it was, thus, increased *in vitro* and *in vivo* by the increased frataxin expression.

INTRODUCTION

Friedreich ataxia (FRDA) is an autosomal recessive hereditary disease, affecting 1 person in 29,000, due to a mutation of the *FXN* gene located on chromosome 9.[1–3] The major observed mutation is a GAA repeat expansion in the intron 1,[4–6] which causes transcriptional silencing due to the formation during RNA elongation of abnormal structures between DNA and RNA at the site of GAA repeats in intron 1, due to different DNA epigenetic changes (such as DNA methylation and histone modifications), and due to heterochromatin formation (by hypoacetylation of histones, mainly histones 3 and 4, and trimethylation of histone 3 lysine 9).[4,5,7,8] As a result, the promoter becomes inaccessible to transcription factors, leading to a severe deficiency of transcriptional initiation in FRDA,[9,10] which leads to a decrease of the frataxin protein to as low as 4%–29% of normal cell level.[2,4,11,12] Frataxin is a 14-kDa mitochondrial protein[2,13] that plays an important role in iron metabolism and in electron transport by iron-sulfur complexes.[2,5,13] The reduced frataxin expression causes abnormal functioning of mitochondrial enzymes, such as aconitase,[14,15] and perturbation of the iron-sulfur complex biosynthesis. This results in free iron accumulation in mitochondria and increased reactive oxygen species (ROS) production that induces oxidative stress and damage in cells.[1] These disturbances result in several of the most striking symptoms of FRDA, which are disorders of movement coordination, neurodegeneration, a cardiomyopathy, dysregulation of blood glucose with diabetes mellitus, optic atrophy, hearing loss, and sleep apnea.[2,5,13,16,17] These symptoms appear between 10 and 15 years of age and lead to the eventual confinement to a wheelchair,[4] and the average age of death was at 37.5 years.[13,18]

There is currently no curative treatment for FRDA, however, several potential treatments are in development.[19–21] The aim of these potential treatments is to reduce and manage symptoms and slow down the progression of the disease. Our project aimed to increase the expression of frataxin by targeting the promoter with TALE (Transcription Activator-Like Effector) proteins fused with a transcriptional activator (TA) VP64 or p300. The latter recruit transcription factors that allow chromatin remodeling, and the promoter becomes more accessible to transcription factors after epigenetic changes and euchromatin formation. The recruitment of RNA polymerase and initiation of transcription of the *FXN* gene are also increased.[22–25]

TALEs are DNA-binding proteins that contain repeated blocks of 34 amino acids. Two amino acids in positions 12 and 13, called Repeat Variable Diresidues (RVDs), determine which nucleotide is bound by this part of the TALE protein.[26,27] Platinum TALEs contain additional modifications of the amino acids in positions 4 and 30 of the 34 amino acid blocks.[28,29] These modifications were reported to increase their DNA-binding affinity.[28,29] TALE proteins were fused with a TA (e.g., VP64 or p300) or with a peptide containing 10 or 24 SunTag (ST) epitopes, each allowing the recruitment of one scFv-VP64 to

Received 28 March 2018; accepted 24 April 2018;
https://doi.org/10.1016/j.omtn.2018.04.009.

Correspondence: Jacques P. Tremblay, Centre de Recherche du CHU, Québec-Université Laval, Québec, QC, Canada.
E-mail: jacques-p.tremblay@crchul.ulaval.ca

This is an open access article under the CC BY license (http://creativecommons.org/licenses/by/4.0/).

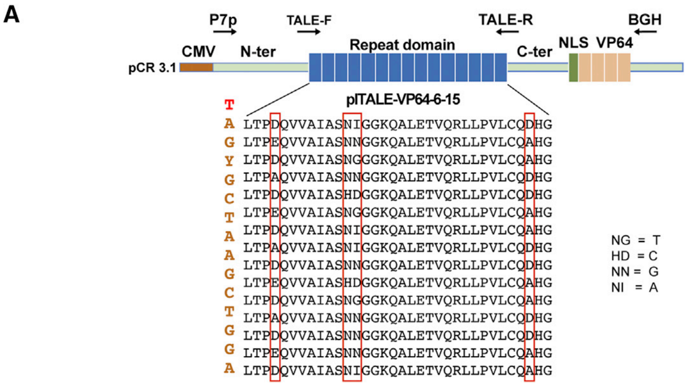

B

pITALE-VP64	pITALE-p300	pITALE-ST10X	pITALE-ST24X	Target sequences	RVD sequences
pITALE-VP64-1-13	-	-	-	TCCCTTGGGTCAGG	HD HD HD NG NG NN NN NN NG HD NI NN NN
pITALE-VP64-2-13	-	-	-	TCCTGGTTGCACTC	HD HD NG NN NN NG NG NN HD NI HD NG HD
pITALE-VP64-3-13	-	-	-	TGCTTTGCACAAAG	NN HD NG NG NG NN HD NI HD NI NI NI NN
pITALE-VP64-6-13	-	pITALE-ST10X-6-13	pITALE-ST24X-6-13	TAGTGCTAAGCTGG	NI NN NG NN HD NG NI NI NN HD NG NN NN
pITALE-VP64-6-15	pITALE-p300-6-15	pITALE-ST10X-6-15	pITALE-ST24X-6-15	TAGTGCTAAGCTGGGA	NI NN NG NN HD NG NI NI NN HD NG NN NN NN NI
pITALE-VP64-7-13	-	pITALE-ST10X-7-13	-	TGGGAAGTTCTTCC	NN NN NN NI NI NN NG NG HD NG NG HD HD
pITALE-VP64-7-15	-	pITALE-ST10X-7-15	-	TGGGAAGTTCTTCCTG	NN NN NN NI NI NN NG NG HD NG NG HD HD NG NN
pITALE-VP64-8-13	-	pITALE-ST10X-8-13	pITALE-ST24X-8-13	TCCTGAGGTCTAAC	HD HD NG NN NI NN NN NG HD NG NI NI HD
pITALE-VP64-8-15	pITALE-p300-8-15	pITALE-ST10X-8-15	pITALE-ST24X-8-15	TCCTGAGGTCTAACCT	HD HD NG NN NI NN NN NG HD NG NI NI HD HD NG
pITALE-VP64-10-13	-	pITALE-ST10X-10-13	-	TGCTCCCCCACAGA	NN HD NG HD HD HD HD HD NI HD NI NN NI
pITALE-VP64-F2-13	-	-	-	TGCCTGCGGCCAGT	NN HD HD NG NN HD NN NN HD HD NI NN NG
pITALE-VP64-11-13	-	-	-	TGGCCACCAGGGGT	NN NN HD HD NI HD HD NI NN NN NN NN NG
pITALE-VP64-F3-13	-	-	-	TGGAGGGCGGAGCG	NN NN NI NN NN NN HD NN NN NI NN HD NN
pITALE-VP64-F4-15	pITALE-p300-F4-15	pITALE-ST10X-F4-15	-	TGCGCAGGGAGGCGCC	NN HD NN HD NI NN NN NN NI NN NN HD NN HD HD

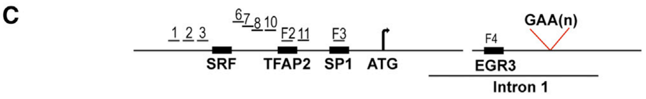

Figure 1. The RVD Sequences of the pITALEs$_{TA}$ and Their Target Sequences

(A) Scheme representing the different molecular parts of the pCR3.1-pITALE$_{VP64}$ (the CMV promoter, N-ter, repeat domain, C-ter, NLS, and VP64) constructs. Primers P7p, TALE-F, TALE-R, and BGH were used for sequencing. (B) List of pITALE$_{VP64}$, pITALE$_{p300}$, pITALE$_{ST10X}$, and pITALE$_{ST24X}$ names with their RVD sequences, which permit binding to the targeted nucleotides. (C) Schematic localization regions 1, 2, 3, 6, 7, 8, 10, 11, F2, F3, and F4 in the promoter and in intron 1 of the *FXN* gene targeted by the pITALEs.

activate the expression of a specific gene.[24,30–32] We have, therefore, produced several platinum TALE-TAs (pITALE$_{VP64s}$, pITALE$_{p300s}$, pITALE$_{ST10Xs}$, and pITALE$_{ST24Xs}$) targeting sequences of 13 or 15 nt in the *FXN* promoter or in intron 1 to activate the expression of that gene. Some of the targeted sequences are the binding sites for transcription factors. The advantage of targeting a longer nucleotide sequence is a potential reduction of off-target effects and an increase in the effectiveness.[33]

Our therapeutic approach was tested *in vitro* in fibroblasts from different FRDA patients by nucleofecting plasmids expressing a pITALE$_{VP64}$ or a pITALE$_{ST}$ under a cytomegalovirus (CMV) or a CMV early enhancer/chicken β-actin (CAG) promoter. We have also tested the efficacy of the best pITALE$_{VP64s}$ and pITALE$_{ST10X}$ *in vivo* by intraperitoneal (i.p.) delivering with an AAV9[34–36] to YG8R mice, an FRDA model.[37–39] These effectors increased the expression of frataxin up to 10-fold in the heart and up to 35-fold in the muscles of treated mice. The development of this technology may also be useful to increase gene expression in other hereditary diseases.

RESULTS

Induction of the *FXN* Gene *In Vitro* in FRDA Fibroblasts with pITALE$_{VP64s}$

We initially constructed 14 pITALE$_{VP64s}$ in vector pCR3.1. These pITALE$_{VP64s}$ targeted 14 sequences of the *FXN* gene (Figures 1A and 1B). We targeted specific sequences of the *FXN* gene regulatory region and the initiation of the transcription, located up to 220 nt upstream of the ATG site (Figure 1C). One of the target sequences (F4) was located at 234 nt downstream of the ATG in intron 1, overlapping the *EGR3* binding site (Figure 1C). Thus, some of the targeted sequences fixed a transcription factor (TFRAP2, SP1, and EGR3; Figure 1C) and others did not. The targeted sequences always started with a 5′ thymidine (T) and were 13 or 15 nt in size, respectively, for pITALE with 13 or 15 RVDs. The number of RVDs may increase the binding efficiency to the target sequence and the efficiency of transcription induction, and it may reduce off-target effects.[33]

The efficacy of each pITALE$_{VP64}$ was tested on FRDA4078 primary fibroblasts (derived from a Friedreich patient with 541/420 GAA repeats) by nucleofection using the same parameters as for the GFP control (the plasmid L6V5 of 9,400 pb) (Figure S1C). These *in vitro* treatments were intended to measure the activity of these effectors on the increase of transcription and expression of frataxin in FRDA4078 cells 3 days after the nucleofection compared to two negative controls. These controls were cells nucleofected only with the nucleofection solution or cells nucleofected with the empty pCR3.1 vector that did not express a pITALE. The pITALE$_{VP64}$ activities were measured by quantifying the number of *FXN* mRNAs per microgram of total RNA extracted from the FRDA4078 cells (Figure 2A). The *FXN* mRNA results were normalized with glyceraldehyde-3-phosphate dehydrogenase (*GAPDH*) mRNAs (Figure 2B). The pITALE$_{VP64s}$ containing 13 RVDs that targeted the sequences 1, 2, 3, 7, F2, 11, and F3 did not increase the *FXN* gene expression of FRDA4078 cells compared to the negative controls. The pITALE$_{VP64s}$, which targeted the sequences 6, 7, and 8 located between the binding sites of the transcription factors SFR and TFAP2, increased the *FXN* gene expression more than other pITALE$_{VP64s}$ that targeted the *FXN* promoter (Figures 2A and 2B). The pITALE$_{VP64s}$ targeting sequences 6 and 8 increased expression by up to 2-fold (****$p < 0.0001$). The pITALE$_{VP64}$, which targeted the F4 sequence, increased *FXN*

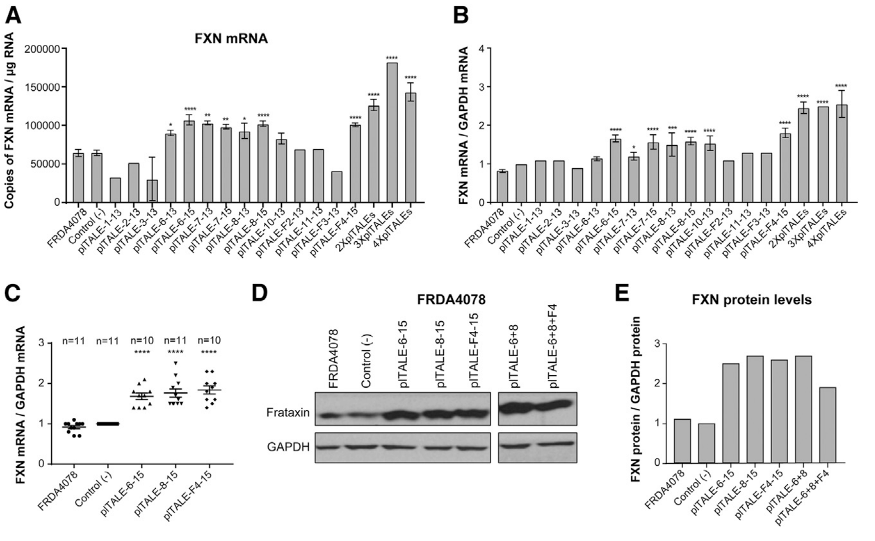

Figure 2. Induction by plTALE$_{VP64s}$ of the *FXN* Gene in FRDA4078 Fibroblasts

(A) Number of *FXN* mRNA copies per microgram of total RNA extracted from cells treated with 14 different plTALE$_{VP64s}$ compared to negative controls. 2×plTALEs is for the result of plTALE$_{6-15}$ with plTALE$_{8-15}$; 3×plTALEs for plTALE$_{6-15}$ + plTALE$_{8-15}$ + plTALE$_{F4-15}$; 4×plTALEs for plTALE$_{6-15}$ + plTALE$_{8-15}$ + plTALE$_{F4-15}$ + plTALE$_{10-15}$. (B) Increased transcriptional activity of the *FXN* gene normalized to *GAPDH* gene transcription in treated FRDA4078 cells compared to negative controls. (C) Induction of the *FXN* gene by the 3 best plTALE$_{VP64s}$ normalized to *GAPDH* gene and compared to the controls (n = 10). (D and E) Induction of expression of the frataxin protein in FRDA4078 cells treated with the best plTALE$_{VP64}$s (alone or in pairs). Western blots for frataxin protein (D) were quantified densitometry and normalized with the GAPDH band (E). *p < 0.05, **p < 0.003, ***p < 0.0003, and ****p < 0.0001. (A and B) Results are the average ± SEM.

mRNA in FRDA4078 cells by more than 2-fold (****p < 0.0001). Thus, plTALE$_{VP64}$ effectors activated up to 2.5-fold the endogenous *FXN* gene in FRDA cells depending on the target sequence and the number of RVDs. The plTALEs with 15 RVDs were more effective than plTALEs with 13 RVDs that targeted the same sequences of the regulatory regions of the FXN gene (Figures 2A and 2B).

The efficacy of the 3 best plTALE$_{VP64}$ (plTALE$_{6-15}$, plTALE$_{8-15}$, and plTALE$_{F4-15}$) to increase the transcription of the *FXN* gene in the FRDA4078 cells was tested ten times (n = 10, ****p < 0.0001) and compared with the negative controls (Figure 2C). The results showed that the 3 plTALE$_{VP64s}$ induced the transcriptional activity of the *FXN* gene with only small efficiency variations. These 3 effectors also increased by 2.5- to 2.7-fold relative to the negative controls the expression of the frataxin protein normalized with GAPDH protein (Figures 2D and 2E).

FXN Gene Transcription with plTALE-STs Recruiting 10 or 24 Copies of VP64

We subsequently made new plTALEs fused with a 10× ST or 24× ST (plTALE$_{ST10Xs}$ or plTALE$_{ST24Xs}$) (Figure 3A), which can recruit 10 or 24 scFv-sfGFP-VP64-GB1 (scFv) (Figure 3B) to further improve the transcription of the endogenous *FXN* gene in FRDA cells.

The *FXN* transcription induced by the 12 plTALE$_{STs}$ in FRDA4078 was normalized with *GAPDH* or hypoxanthine phosphoribosyltransferase (*HPRT*) mRNAs and compared to the negative controls (the untreated cells and the cells treated with the vector expressing scFv and the empty vector pCR3.1) (Figure 3C). The plTALE$_{ST10Xs}$ (containing 10 STs) were more effective at increasing frataxin expression than plTALE$_{ST24Xs}$ (containing 24 STs) targeting the same sequence (Figure 3C; see also Figure S4). Indeed plTALE$_{ST24X-6-15}$ and plTALE$_{ST24X-8-15}$ (containing 24 STs and 15 RVDs and targeting, respectively, sequences 6 and 8) increased significantly *FXN* transcription in FRDA4078 cells by about 2-fold (Figure 3C, left panels), whereas plTALE$_{ST10X-6-15}$ and plTALE$_{ST10X-8-15}$ significantly increased *FXN* transcription by 4- to 5-fold (Figure 3C, right panels). These results also confirmed that plTALE$_{ST10Xs}$ containing 15 RVDs were more effective than those containing 13 RVDs. The two best plTALE$_{ST10Xs}$ (plTALE$_{ST10X-6-15}$ and plTALE$_{ST10X-8-15}$) also increased the expression of the frataxin protein in FRDA4078 cells 2 days after the treatment by about 3-fold (Figures 3D and 3E). None of the plTALE$_{ST24Xs}$ and the plTALE$_{ST10X-10-13}$ increased expression of the frataxin in FRDA4078 cells (Figures 3D and 3E).

FRDA4078 cells were treated with 2 plTALE$_{ST10X}$ together containing either 13 or 15 RVDs targeting sequences 6 and 8 (Figures 4A and 4B). The 2 plTALE$_{ST10Xs}$ containing 13 RVDs increased *FXN* transcription about 3-fold, whereas the two plTALE$_{ST10Xs}$ containing 15 RVDs had a stronger synergic effect and increased *FXN* transcription more than 11-fold (****p < 0.0001), resulting in a 5-fold increase of the frataxin protein (Figures 3D and 3E). Thus, the combination of two plTALE$_{ST10Xs}$ with 15 RVDs targeting sequences 6 and 8, which are separated by 10 nt, recruited up to 20 VP64s at those target regions located between the SFR and TFAP2 sites, resulting in a strong transcription activity of the *FXN* gene.

Reversible modulation of aconitase has been used as a biomarker of FRDA oxidative stress;[40,41] the aconitase activity may thus be used as an indicator of the activity of frataxin in the mitochondria. The activity of aconitase was measured in cells treated with plTALE$_{ST10X-6-15}$ plus plTALE$_{ST10X-8-15}$ together and negative controls (untreated cells and cells treated with pCR3.1-scFv plus pCR3.1 empty) (Figure 4C). The aconitase activity was increased significantly by more than 2.2-fold in the cells treated with these effectors compared to the negative controls (n = 3, *p = 0.03). Thus, these results indicated that the increase in transcription of the endogenous *FXN* gene and the resulting expression of the frataxin protein in the cells treated with the plTALE$_{ST10Xs}$ increased aconitase activity. Therefore, they strongly suggest that plTALE$_{ST10Xs}$ can correct the molecular and biochemical symptoms of FRDA.

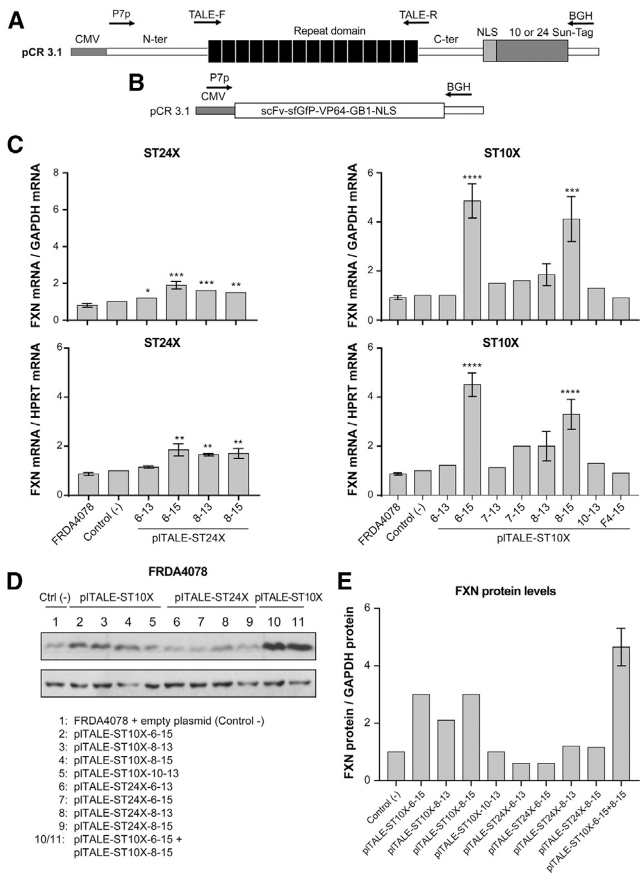

Figure 3. Treatment of FRDA4078 Fibroblasts with plTALE$_{ST10Xs}$ and plTALE$_{ST24Xs}$

(A) Scheme of pCR3.1-plTALE$_{ST}$ expressing a plTALE fused with 10 or 24 SunTag (ST) epitopes, each recruiting an scFv-sfGFP-VP64. (B) Scheme of pCR3.1-scFV-sfGFP-VP64-GB1-NLS, which expresses an scFv binding with an ST to activate the expression of the *FXN* gene. (C) Induction of *FXN* gene expression in FRDA4087 cells by a plTALE$_{ST10X}$ or a plTALE$_{ST24X}$ normalized with *GAPDH* and *HPRT* mRNAs compared to FRDA4078 controls. The plTALE$_{ST24Xs}$ slightly increased *FXN* gene expression in FRDA4078 cells. This increase reached about 2-fold for plTALE$_{ST24X\text{-}6\text{-}15}$ normalized by *GAPDH* or *HPRT*. The plTALE$_{ST10X\text{-}6\text{-}15}$ and plTALE$_{ST10X\text{-}8\text{-}15}$ induced *FXN* transcription normalized with *GAPDH* by 4- to 5-fold. (D and E) Frataxin protein in negative controls and in the cells treated with 1 or 2 plTALE$_{ST10X}$ or with 1 plTALE$_{ST24X}$ normalized with GAPDH protein. Western blots for frataxin protein (D) were quantified by densitometry and normalized with the GAPDH band (E). $^{*}p < 0.05$, $^{**}p < 0.003$, $^{***}p < 0.0003$, and $^{****}p < 0.0001$. (C and E) Results are the average ± SEM.

The plTALE$_{ST}$ recruits scFv-sfGFP-VP64-GB1 fluorescent proteins. Thus, the fluorescent label is concentrated in the nucleus when a plTALE$_{ST}$ is present (see the Supplemental Results).

Treatment of Cell Lines from 4 FRDA Patients with Selected plTALE$_{VP64s}$ and plTALE$_{ST10Xs}$

The number of GAA repeats varies from patient to patient. This variation influences the transcriptional activity of the *FXN* gene and, therefore, the level of expression of the frataxin protein. We treated the primary fibroblasts of 4 FRDA patients with the 3 best plTALE$_{VP64}$ effectors and the 2 best plTALE$_{ST10X}$ effectors previously selected to test the effectiveness of this therapeutic approach. These fibroblasts contained different numbers of GAA repeats (i.e., FRDA66 cells, 240/640; FRDA162 cells, 355/805; FRDA4675 cells, 255/1,140; and FRDA4743 cells, 470/970). They were treated with the same nucleofection protocols previously used to treat the FRDA4078 cells.

The activity of each plTALE$_{VP64}$ on each FRDA cell type was normalized with *GAPDH* or *HPRT* mRNAs and compared to 2 negative controls (untreated cells and cells treated with an empty vector pCR3.1) (Figure 5). We detected significant positive effects in the 4 FRDA cell types treated with the 3 plTALE$_{VP64s}$. The treatment increased the number of copies of *FXN* mRNA approximately 2-fold in the 4 FRDA cell types (Figure 5A). The transcriptional activity of frataxin was normalized with *GAPDH* or *HPRT* mRNAs, and an increase of about 1.7- to 2.5-fold was obtained in FRDA66 and FRDA4675 and between 1.6- and 2.1-fold in FRDA162 and FRDA4743 by the 3 plTALE$_{VP64s}$ (Figures 5B and 5C). These results show that the 3 plTALE$_{VP64s}$ (i.e., plTALE$_{VP64\text{-}6\text{-}15}$, plTALE$_{VP64\text{-}8\text{-}15}$, and plTALE$_{VP64\text{-}F4\text{-}15}$) induced *FXN* transcription in FRDA cells having different numbers GAA repeats. Thus, these plTALE$_{VP64s}$ were effective even when a large number of GAA repeats were present, causing epigenomic silencing and a decrease in the transcription of the *FXN* gene FRDA4743 and FRDA162 (Figure 5A).

We analyzed the efficacy of previously selected plTALE$_{VP64s}$ to induce the expression of frataxin protein in the cells of two FRDA patients with a large number of repeats, FRDA4675 with 255/1,140 repeats (Figures 5D and 5E) and FRDA4743 having 470/970 repeats (Figures 5F and 5G). The plTALE$_{VP64}$ effectors increased the expression of the frataxin protein of the treated FRDA cells. Increases of about 1.5- to 2-fold were obtained in FRDA4675 and FRDA4743 treated with plTALE$_{VP64\text{-}6\text{-}15}$, plTALE$_{VP64\text{-}8\text{-}15}$, and plTALE$_{VP64\text{-}F4\text{-}15}$.

The efficacy of the 2 best plTALE$_{ST10Xs}$ was normalized with *GAPDH* or the *HPRT* mRNAs and compared to negative controls (untreated cells and cells treated with pCR3.1 empty and pCR3.1-scFv) and the different NED (healthy human fibroblast) cell lines (Figure 6A). The two plTALE$_{STs}$ alone or in association activated significantly the transcription of the *FXN* gene in the 4 treated FRDA cells when compared with the negative controls and with the cells of 4 normal subjects. The effects of plTALE$_{ST10Xs}$ increased the transcription of the endogenous *FXN* gene in FRDA66 cells (Figures 6B and 6C), by 5.5-fold by plTALE$_{ST10X\text{-}6\text{-}15}$ and more than 4.2-fold by plTALE$_{ST10X\text{-}8\text{-}15}$, with a synergistic effect of these 2 plTALE$_{ST10Xs}$ in these cells by 14.6-fold. In the FRDA162 cells (Figures 6B and 6C), the effect of plTALE$_{ST10X\text{-}6\text{-}15}$ increased *FXN* transcription more than 3-fold. PlTALE$_{ST10X\text{-}8\text{-}15}$, on the other hand, increased the *FXN* transcription by up to 3.6-fold. These 2 plTALE$_{ST10Xs}$ showed a synergic effect and increased *FXN* transcription by more than 10-fold in the FRDA4078 cell line. In the FRDA4675 cells (Figures 6B and 6C), plTALE$_{ST10X\text{-}6\text{-}15}$ and plTALE$_{ST10X\text{-}8\text{-}15}$ increased

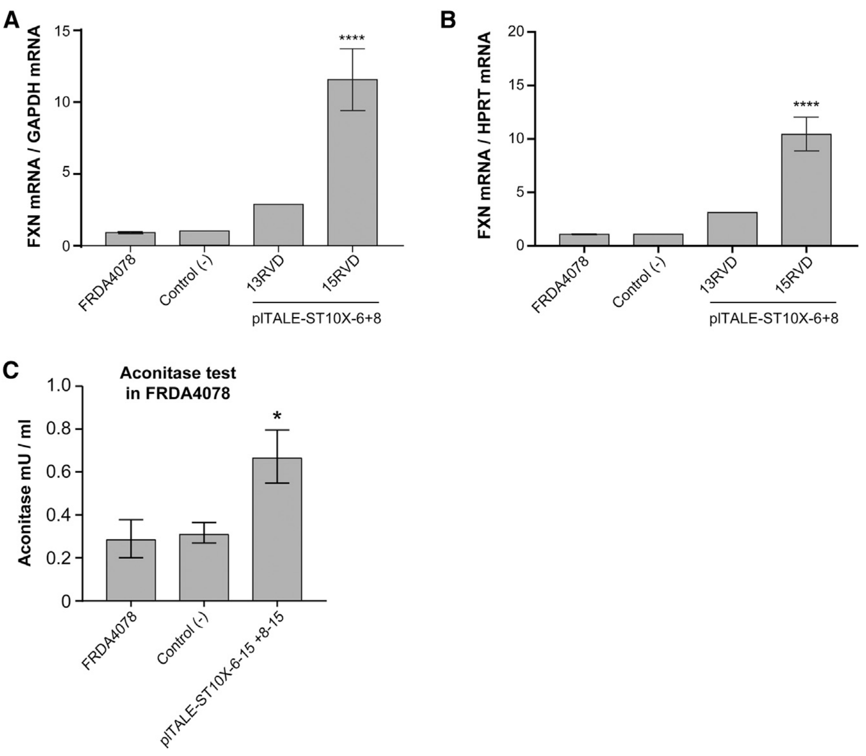

Figure 4. The Synergistic Effect of plTALE$_{ST10x}$ on the Transcription of Endogenous *FXN* Gene and on Aconitase Activity

(A and B) The synergistic effect of 2 plTALE$_{ST10Xs}$ (targeting sequences 6 and 8) containing either 13 or 15 RVDs on the activation of *FXN* gene transcription in FRDA4078 cells. The number of copies of *FXN* mRNAs was normalized with *GAPDH* (A) or *HPRT* (B) mRNAs (n = 5). (C) Increased aconitase activity in FRDA4078 cells treated with plTALE$_{ST10X-6-15}$ + plTALE$_{ST10X-8-15}$ plasmids. This increase is relative to negative controls (untreated FRDA4078 cells) and negative cells (control−) treated with pAAV-scFV-sfGFP-VP64-GB1-NLS and with pCR3.1 empty plasmid (n = 3). *p < 0.05 and ****p < 0.0001. (A–C) Results are the average ± SEM.

FXN transcription normalized with *GAPDH* more than 4-fold, and their synergistic effect increased the *FXN* transcription in the FRDA4675 cells more than 19-fold. Each plTALE$_{ST10X-6-15}$ and plTALE$_{ST10X-8-15}$ separately increased *FXN* transcription in FRDA4743 cells (Figures 6B and 6C) about 3.5-fold. These 2 effectors had a synergic effect and increased by 12-fold *FXN* expression. These results showed that plTALE$_{ST10X-6-15}$ and plTALE$_{ST10X-8-15}$ were effective even in cells (FRDA162, FRDA4675, and FRDA4743) with a large number of GAA repeats and, thus, with a low frataxin expression.

Proteins from 4 different FRDA patient cells were analyzed by western blots after treatment with plTALE$_{ST10X}$ (Figures 6D and 6E). PlTALE$_{ST10X-6-15}$ and plTALEs$_{ST10X-8-15}$ alone increased the frataxin protein by 7.5- and 9.7-fold, respectively, in FRDA66 and by 3- to 4.8-fold in FRDA162, FRDA4675, and FRDA4743. Frataxin expression was much stronger with the synergistic effect of these two effectors in the FRDA66 and FRDA4675 cells, the increase in fact exceeding 12-fold. In conclusion, the strong activation of the transcription of the *FXN* gene of the FRDA cells having different numbers of GAA repeats also increased the expression of the frataxin protein in these cells treated by these effectors.

These results clearly indicated that plTALE$_{VP64}$ and plTALE$_{ST}$ effectors that target specific sequences can activate the transcription of the endogenous *FXN* gene as well as the expression of the frataxin protein of the treated cells.

Induction of the *FXN* Gene *In Vivo* in YG8R Mice by plTALE$_{VP64}$ Effectors

The 3 best plTALE$_{VP64s}$ (i.e., plTALE$_{6-15}$, plTALE$_{8-15}$, and plTALE$_{F4-15}$) were tested *in vivo* in YG8R mice,[37–39] an FRDA model. The mice were injected i.p. between 7 and 11 days of age with doses of AAV9-plTALE$_{VP64}$ ranging from 6×10^{11} and 18×10^{11} viral particles (v.p.). The negative controls were YG8R mice not treated or treated with an AAV9 not expressing the plTALE$_{VP64}$ (AAV-Ctrl). During treatment, the body weight of the treated mice was similar to the body weight of the untreated mice (Figure S5). The mice were sacrificed 1 month after injection of the AAV9 to verify the effects of these AAV9-plTALE$_{VP64s}$ on the activation of the *FXN* gene. RNA, DNA, and proteins were extracted from different tissues (muscles, heart, liver, and brain).

The presence of the virus was detected by qPCR in all tested tissues (muscles, heart, liver, and brain) but predominantly in the heart (Figure S6A, left). No virus was detected in the untreated YG8R mice. The amount of viral genome detecetd varied proportionally to the dose of injected AAV9 expressing the plTALE$_{VP64}$. The distribution of AAV9-plTALE$_{VP64s}$ in the different organs influenced the expression of plTALE$_{VP64}$ effectors in these organs (Figure S6B). A higher expression was detected in the heart (Figure S6B, left) than in the other organs (Figure S6B on the right). However, the plTALE$_{VP64}$ expression depended not only on the amount of virus present in the organ but also on the nature of the organ. Indeed, in the liver, the number of v.p. detected was similar to that in the muscle (Figure S6A, right), but the TALE expression was 10-fold higher in the muscle than in the liver (Figure S6B, right).

A 2-fold significant increase of transcriptional activity of the *FXN* gene was observed in the heart of mice injected with 6 × 1011 or 18×10^{11} AAV9-plTALE$_{VP64-8}$ (Figure 7A). A small non-significant increase was also detected with the AAV9, which expressed plTALE$_{VP64-F4}$, compared to untreated mice. In the muscles of the

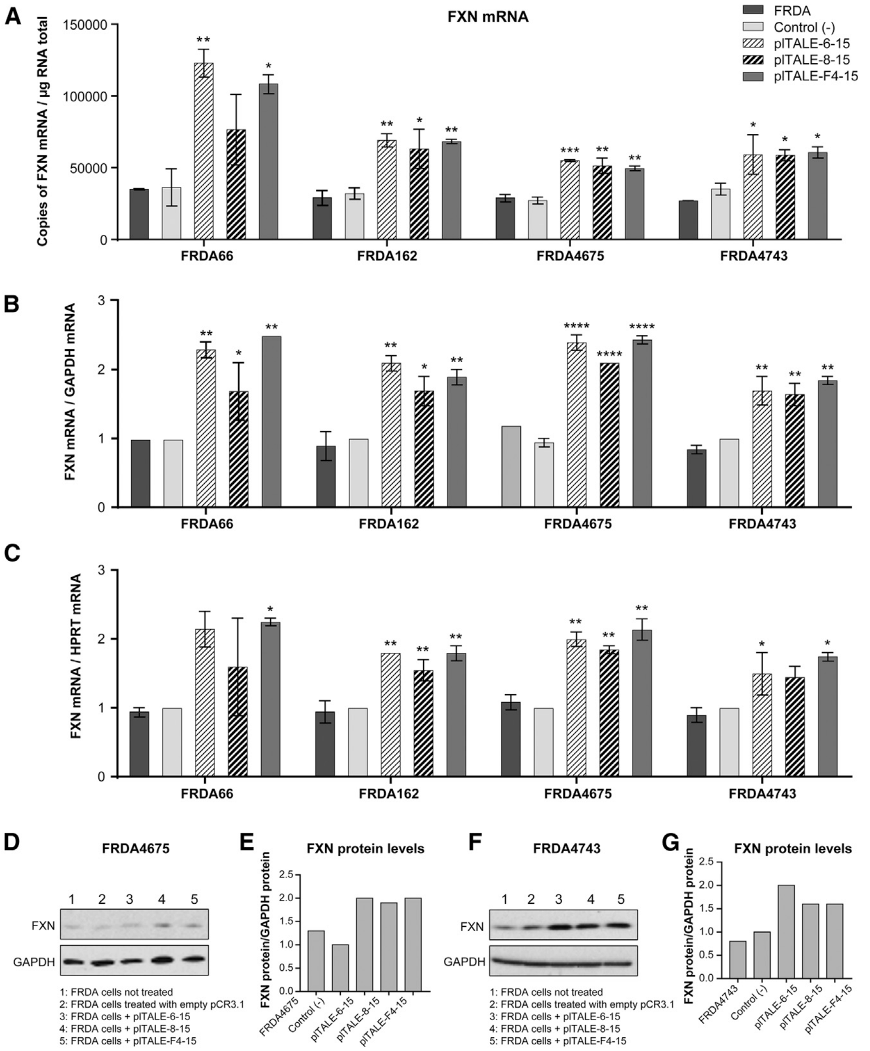

Figure 5. Induction of the *FXN* Gene in 4 Different Types of FRDA Cells with 3 Best plTALE$_{VP64}$

Fibroblasts of 4 different FRDA patients (i.e., FRDA66, with 240 and 640 GAA repeats; FRDA162, 355/805; FRDA4675, 255/1,140; and FRDA4743, 470/970) were treated with plTALE$_{VP64\text{-}6\text{-}15}$, plTALE$_{VP64\text{-}8\text{-}15}$, or plTALE$_{VP64\text{-}F4\text{-}15}$ to increase the expression of the endogenous *FXN* gene. The *FXN* expression was compared to untreated cells and cells treated with empty pCR3.1. (A) The expression of the *FXN* gene in control cells and in treated FRDA cells is presented as the number of *FXN* mRNA copies per microgram RNA. (B and C) The *FXN* mRNAs are normalized with either *GAPDH* (B) or *HPRT* (C) mRNAs. (D–G) Frataxin protein in cells from these 2 FRDA (D and E in FRDA4675 and F and G in FRDA4743) patients were treated with the best plTALE$_{VP64s}$ and compared with negative controls (untreated cells or cells treated with empty plasmid). *$p < 0.05$, **$p < 0.003$, ***$p < 0.0003$, and ****$p < 0.0001$. (A–C) Results are the average ± SEM.

mice treated with AAV9-plTALE$_{VP64}$, the transcriptional activity was significantly increased with AAV9-plTALE$_{VP64\text{-}8}$ by approximately 2-fold with the 18×10^{11} dose and 1.5-fold with the 6×10^{11} dose compared to negative controls. Transcription of the *FXN* gene was also increased in the muscles of mice treated with 6×10^{11} or 18×10^{11} AAV9-plTALE$_{VP64\text{-}6}$, respectively, by 1.4- and 1.7-fold (significant increases). In contrast, the transcriptional activity of the *FXN* gene did not change in the liver and in the brain of mice treated with most of the AAV9-plTALE$_{VP64s}$. However, a small increase in transcription of the *FXN* gene was observed in the liver of the mice treated with AAV9-plTALE$_{VP64\text{-}8}$ and in the brain of the mice treated with AAV9-plTALE$_{VP64\text{-}6}$ (18×10^{11} v.p.). These results strongly suggest that the variation in virus distribution from one organ to another affected the expression of plTALE$_{VP64}$ effectors but the tissue specificity also affected the expression.

The increase in the expression of the frataxin protein in these organs also depended on the increase in the transcription of the *FXN* gene (Figure 7B). In the heart, the expression of frataxin was increased 2.4-fold (significant) and 1.8-fold (not significant) with AAV9-plTALE$_{VP64\text{-}6}$ with the doses 6×10^{11} and 18×10^{11} v.p., respectively. Increases of 2-fold (not significant) and 2.1-fold (significant) were obtained with AAV9-plTALE$_{VP64\text{-}8}$ at doses of 6×10^{11} and 18×10^{11} compared to the untreated mice. In the muscles of the treated mice, the quantity of frataxin protein was increased by 2-fold and 4.6-fold with AAV9-plTALE$_{VP64\text{-}8}$ of the doses 6×10^{11} and 18×10^{11} and by 1.5-fold and 3.5-fold with AAV9-plTALE$_{VP64\text{-}6}$ of the doses 6×10^{11} and 18×10^{11} v.p., respectively. The increases with the higher dose (18×10^{11} v.p.) were significant compared to the untreated mice. The frataxin protein did not increase in other organs (liver and brain).

Induction of the Human *FXN* Gene in the YG8R Mice with plTALE$_{ST10Xs}$

The efficacy of the best plTALE$_{ST10Xs}$ was tested *in vivo* in YG8R mice. The mice were injected i.p. either with AAV9-plTALE$_{ST10X\text{-}6}$ + AAV9-scFv, with AAV9-plTALE$_{ST10X\text{-}8}$ + AAV9-scFv or with AAV9-plTALE$_{ST10X\text{-}6}$ + AAV9-plTALE$_{ST10X\text{-}8}$ + AAV9-scFv. We used doses of 1.5×10^{11} v.p. (0.25× dose), 3×10^{11} v.p. (0.5× dose), or 6×10^{11} v.p. (1× dose). The fluorescent label distribution in the treated mice was compared with the negative controls, i.e., untreated mice and mice treated with AAV9-scFv and an AAV9 that did not express a plTALEs$_{ST10X}$ (named Ctrl−). The confocal image of the tissues (muscles and heart) of treated mice with the AAV9s expressing a plTALE$_{ST}$ and an scFv showed the location of the sfGFP fluorescence in the nuclei (Figures S7A and S7B). The intensity of the sfGFP fluorescence was much brighter in the nuclei of tissues treated with AAV9

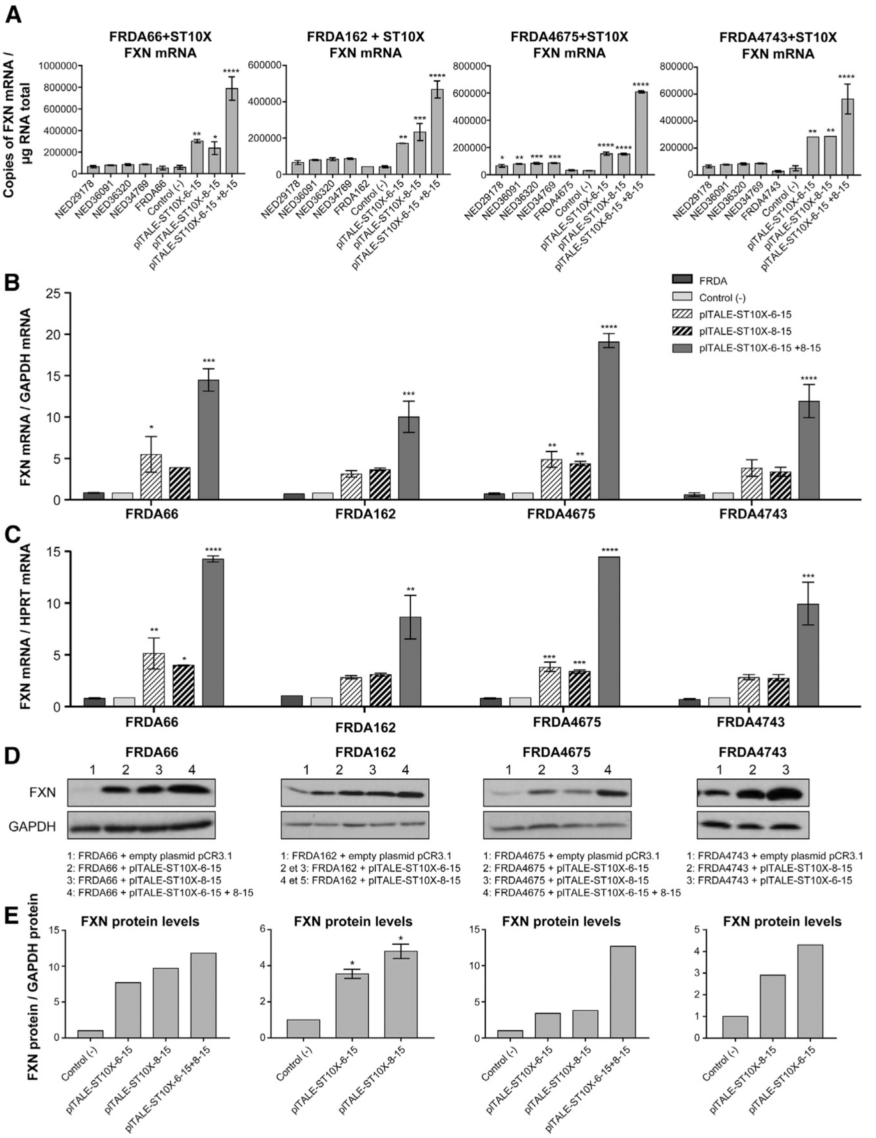

Figure 6. Induction of the *FXN* Gene by the 2 Best plTALE$_{ST10X}$ and Their Synergistic Effect in Fibroblasts of 4 Different FRDA Patients Compared with Those of 4 Normal Subjects

(A) Number of copies of *FXN* mRNA in 4 different normal fibroblasts (NED) and in 4 FRDA cells (FRDA66 cells, with 240/640 GAA repeats; FRDA162 cells, with 355/805 GAA repeats; FRDA4675 cells, with 255/1,140 GAA repeats; and FRDA4743 cells, with 470/970 GAA repeats) in negative control fibroblasts and after induction of the endogenous *FXN* gene with the 2 best plTALE$_{STs}$ (i.e., plTALE$_{ST10X-6-15}$ and plTALE$_{ST10X-8-15}$). (B and C) *FXN* mRNA normalized with *GAPDH* (B) or *HPRT* (C) mRNAs in various FRDA cells not treated or treated with plTALE$_{ST10X-6-15}$, plTALE$_{ST10X-8-15}$ alone or in pair. (D and E) Induction of the expression of the frataxin protein in cells from these 4 FRDA patients treated with 1 or 2 plTALE$_{ST10X}$ with respect to the negative controls. Western blots for frataxin protein (D) were quantified by densitometry and normalized with the GAPDH band (E). *$p < 0.05$, **$p < 0.003$, ***$p < 0.0003$, and ****$p < 0.0001$. (A–C and E) Results are the average ± SEM.

expressing the scFv-sfGFP-VP64 (scFv) and the effectors plTALE$_{ST10X-6}$ and plTALE$_{ST10X-8}$ than in tissues expressing only the scFv. These two plTALE$_{STs}$ were able to recruit together up to 20 scFv, thereafter allowing the localization of the fluorescent label (scFv-sfGFP-VP64) complexes in target sites in the *FXN* gene promoter.

The detection of virus particles was made in different organs: muscles, heart, liver, and brain. Both viruses were detected, i.e., the one containing the sfGFP (Figure S8A) and the one containing the plTALE$_{ST}$ (Figure S8B). For both viruses, more copies were detected in the heart than in the other organs (Figures S8A and S8B). Fewer viruses were detected in the brain probably in part due to the blood-brain barrier. The expressions of sfGFP (Figure S8C) and of plTALE$_{ST}$ (Figure S8D) were both higher in the heart, followed by the muscle, and again the lowest in the brain.

The transcriptional activity of the *FXN* gene in the muscle, and in the heart of mice treated with a single plTALE$_{ST10X}$ or with the combination of the two plTALE$_{ST10Xs}$ together, was strongly activated (Figure 8A). The transcriptional activity increased in mice treated with the plTALE$_{ST10X-6}$ or with the plTALE$_{ST10X-8}$ by 5.7- and 5.3-fold, respectively, in the muscles and by 2- to 3.5-fold in the heart. The combination of two different plTALE$_{ST10Xs}$ (3×10^{11} v.p. of plTALE$_{ST10X-6}$ + 3×10^{11} v.p. of plTALE$_{ST10X-8}$) produced additive significant effects on the transcription of the *FXN* gene, and it resulted in an 11.8-fold increase in the muscle and a 5.5-fold increase in the heart compared to the negative controls. The *in vivo* synergistic effect of AAV9-plTALE$_{ST-6}$ (1.5×10^{11} v.p.) and AAV9-plTALE$_{ST-8}$ (1.5×10^{11} v.p.) significantly increased transcription by 21.6-fold in the muscles and by 4.5-fold in the heart of the treated YG8R mice. There was only a small increase of transcription in the liver and no effect in the brain. Strong increases of the frataxin protein were observed in the muscles and the heart of mice treated with these plTALE$_{ST10Xs}$ compared to the negative controls (Figure 8B). These increases were 8-fold and 3.3-fold in the muscles and 2.8- to 3.5-fold in the heart of mice treated with plTALE$_{ST10X-6}$ or with plTALE$_{ST10-8}$, respectively. An additive effect was observed when both effectors (i.e., AAV9-plTALE$_{ST-6}$ and AAV9-plTALE$_{ST-8}$) were used together; the increase reached 15-fold in the muscle and 6-fold (significant) in the heart. In the liver, a significant increase was also detected, ranging between 2.4- and 3-fold compared to the untreated mice. However, the synergistic effect of these 2 effectors significantly

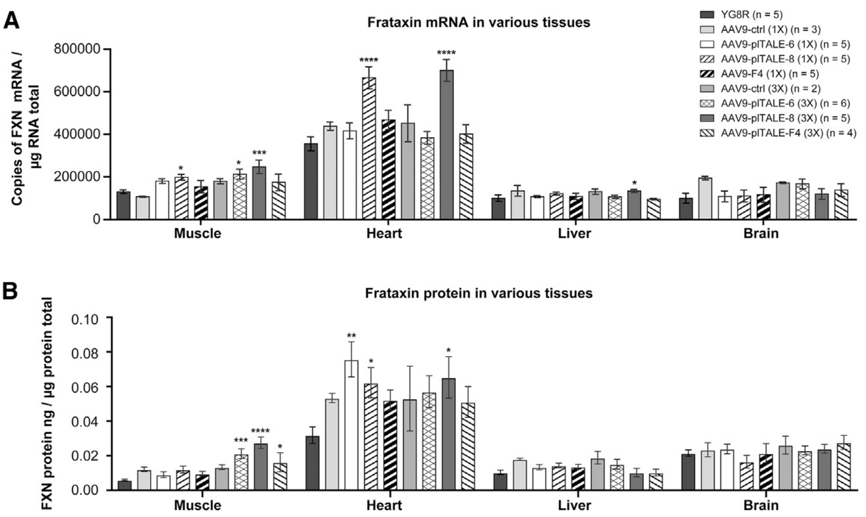

Figure 7. *In Vivo* Induction of the *FXN* Gene by AAV9-plTALE$_{VP64s}$

The *FXN* gene was induced in mice treated with AAV9, which expressed either the plTALE$_{6\text{-}15}$, plTALE$_{8\text{-}15}$, or plTALE$_{F4\text{-}15}$. The *FXN* gene expression was analyzed in four organs (muscle, heart, liver, and brain). The negative controls YG8R mice were either untreated or treated with AAV9 (AAV9-ctrl), which did not express plTALE$_{VP64}$. (A) The transcriptional activity of the *FXN* gene. (B) The expression of the frataxin protein in YG8R mice treated with plTALE$_{VP64}$ compared with negative control YG8R mice. *$p < 0.05$, **$p < 0.003$, and ****$p < 0.0001$. (A and B) Results are the average ± SEM.

increased the expression of frataxin protein by 35-fold in the muscles, 10-fold in the heart, 3-fold in the liver, and 2-fold in the brain (Figure 8B). These strong inductions of the expression of frataxin with the synergistic effect increased the activity of the aconitase in the heart of the treated mice compared with the untreated mice (Figure 8C).

This induction of the *FXN* gene makes it possible to regulate the expression of the other genes, e.g., *PGC-1α* and *GAD65*, which are necessary for mitochondrial biogenesis. Expression of these genes is regulated with the level of expression of the frataxin protein in cells.[42] The expression of PGC-1α was increased (Figures 9B and 9E) when the expression of frataxin increased (Figures 9A and 9D) in mice treated with AAV9-plTALE$_{ST10X}$. This increase was significant in the muscles (Figure 9B) of the treated mice, but not in the heart (Figure 9E). *FXN* gene activation decreased the expression of the *GAD65* gene significantly (Figure 9C) in the muscles of the treated mice, and the increase did not reach a significant level in the heart (Figure 9D). These results strongly suggest that the plTALE$_{ST10X}$ system can restore FRDA phenotypes *in vivo* in FRDA model mice. This encourages us to continue the development of this plTALE$_{TA}$ system to aleviate cardiac and neurological FRDA symptoms.

DISCUSSION

The improved understanding of the FRDA molecular pathogenesis[4,5,7–10,43] and the TALE technology development[22–25,27,28] have both contributed to the development of our proposed therapeutic approach, which aims to activate the transcription initiation of the *FXN* gene to restore the normal mitochondrial function in FRDA cells.

TALEs are DNA-binding proteins that can be fused with a TA, such as VP64 or p300, to increase endogenous gene expression by the activation of transcription initiation of the target gene.[24,25,31,44] This approach has been used to induce the activation of the *Oct4* (Pou5f1),[31] *IL1RN*, *MYOD*, and *OCT4* genes[24] and to reactivate the latent *HIV-1* provirus.[30] We used plTALEs that are easier to construct and have a higher specificity and affinity toward their target sequences than normal TALEs, as shown by the Tetsushi group.[28] We targeted with 14 plTALE$_{VP64s}$ different sequences in the *FXN* promoter and intron 1 that fix or not transcription factors. The plTALE$_{VP64s}$ targeting the sequences 6, 8 (located between the SFR- and TFAP2-binding sites), and sequence F4 (near the *EGR3* binding site in intron 1)[45] strongly activated the transcription of the *FXN* gene in FRDA cells. This confirmed previous results of our group[46] that a TALE-VP64 containing 13 RVDs and targeting sequence 8 activated the *FXN* gene in FRDA cells. These results showed that the initiation of transcription is very strong at sites 6 and 8. This induced chromatin remodeling and promoter activation after epigenetic change through the recruitment of transcription complexes by plTALE$_{VP64}$.[22–25]

The plTALEs fused with p300-targeting sequences 6, 8, and F4 were less effective than plTALEs fused with VP64 targeting the same sequences (Figure S2D), thus confirming the results of Hilton et al.[24] for *IL1RN*, *MYOD*, and *OCT4* genes. Anthony et al.[47] reported a synergistic effect when a TALE fused with the TATA-box-binding protein (TBP-TALE) and a TALE$_{VP64}$ were used together for the activation of the *IL-2* gene in 293T cells. However, in our experiments, the combination of the plTALE$_{VP64}$ and plTALE$_{p300}$ did not produce a synergistic effect on *FXN* gene transcription (Figure S2E).

Treatment of FRDA4078 cells with two p1TALE$_{p300s}$ targeting sequences 6-15 and 8-15 increased the transcription and expression of the *FXN* gene by about 2-fold (Figures S3A and S3B). This result is the equivalent of the effect of a single plTALE$_{VP64}$ targeting sequences 6-15, 8-15, or F4-15. This suggests that the acetylation of chromatin by the p300 activator only occurs when the target sequences are far away from the transcription initiation site. Our results confirm those of Hu et al.,[31] who targeted the Oct4 gene enhancer region with p300. However, the high methylation of the FRDA *FXN* gene can be a major obstacle to acetylation of this gene by p300. Xu et al.[48] have used a DNA demethylase (dCas9-MS2-Tet1-CD) to resolve a similar gene-silencing problem.

Ji et al.[49] showed *in vitro* activation of the *HIV-1 LTR* promoter with a dCas9-ST24X. We thus used the plTALE$_{ST}$ system to further increase the transcription activity of VP64 on the endogenous *FXN* gene. An ST

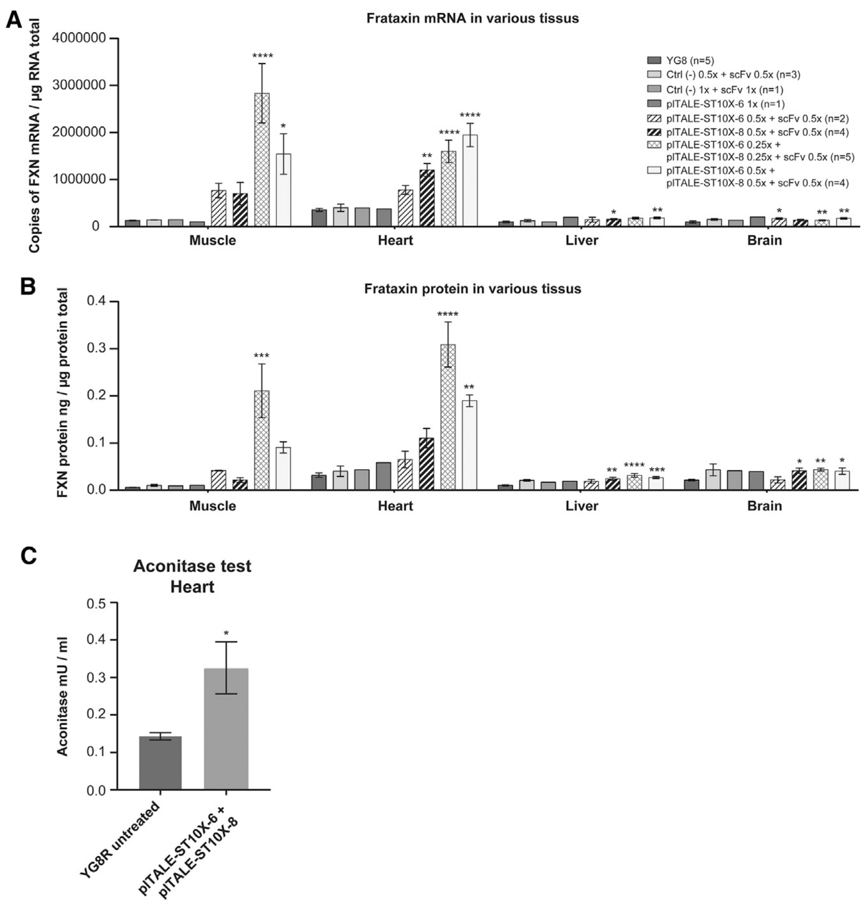

Figure 8. Induction of the *FXN* Gene *In Vivo* in YG8R Mice Treated with AAV9 Expressing the plTALE$_{ST10X-6-15}$ or plTALE$_{ST10X-8-15}$ and AAV9 Expressing scFV-GFP-VP64

Mice were treated with two AAV9s (one containing scFV and the other one plTALE$_{ST10X-6-15}$ or plTALE$_{ST10X-8-15}$). To test potential synergistic effects of the combination of these two effectors, three AAV9 were used (AAV9-scFV, AAV9-plTALE$_{ST10X-6-15}$, and AAV-plTALE$_{ST10X-8-15}$). Several tissues were analyzed (muscle, heart, liver, and brain) in the treated mice and compared to those of control YG8R mice: untreated or treated with an AAV9, Ctrl (−), that did not express plTALE$_{ST10X}$. (A) Quantification of the number of *FXN* mRNAs by qRT-PCR in various organs of 3 different types of control mice and 4 groups of mice treated with the plTALE$_{ST10Xs}$. Additive and synergic effects were observed in muscles and heart when plTALE$_{ST10X-6}$ and plTALE$_{ST-10X-8}$ were used together. (B) The frataxin protein was significantly increased in muscle, heart, and liver of mice that received various plTALE$_{ST10X}$ treatments. (C) Increased aconitase activity in the hearts of mice treated with AAV9-plTALE$_{ST10X-6-15}$ + AAV9-plTALE$_{ST10X-8-15}$ + pAAV-scFv plasmids. This increase is relative to negative controls (untreated mice) (n = 4). $^*p < 0.05$, $^{**}p < 0.003$, $^{***}p < 0.0003$, and $^{****}p < 0.0001$. (A–C) Results are the average ± SEM.

fused with a single plTALE permits recruitment of 10 or 24 VP64s at the regulatory region of the *FXN* gene. Our results showed that a single plTALE$_{ST10X}$ induced a stronger expression of the endogenous *FXN* gene than a plTALE$_{VP64}$. A similar observation was made by Tanenbaum et al.[32] with a dCas9 protein fused with an ST and a guide RNA (gRNA) targeting the promoter of the *CDKN1B* gene. However, the plTALE$_{ST24X}$ and some plTALE$_{ST10X}$ targeting non-active promoter sequences or the *EGR3* binding sequence did not increase the *FXN* gene expression. Moreover, the synergistic effect of these effectors plTALE$_{ST10X}$ reactivated the endogenous *FXN* gene in FRDA cells even more strongly compared to the effect of a single plTALE$_{ST10X}$. In fact, a single plTALE$_{ST10X}$ targeting sequence 6-15 or sequence 8-15 induced the transcription initiation of the *FXN* gene after recruitment of the transcriptional complex by several VP64s (10 VP64 for plTALE$_{ST10X}$).[24,25,27,31,50] Two plTALE$_{ST10Xs}$ targeting two active regions of the transcription initiation site recruited a total of 20 VP64s, allowing a powerful activation of the endogenous *FXN* gene.

The plTALE containing 15 RVDs induced a higher *FXN* expression than the plTALE containing 13 RVDs, which targeted the same sequences. This may be because the higher number of RVDs increased the specificity and the affinity of the plTALE proteins for their DNA target sequences. Our results confirm those of Rinaldi et al.[33]

The efficacies of the best plTALE$_{VP64s}$ and plTALE$_{ST10Xs}$, as potential FRDA treatments, were investigated in fibroblasts of four FRDA patients containing different numbers of GAA repeats. Our results showed that the 3 best plTALE$_{VP64s}$ activated transcription in all FRDA cells, even in cells with a large number of GAA repeats (355/805 and 470/970). A strong synergistic increase of the *FXN* mRNA and of the frataxin protein was obtained when two effectors (plTALE$_{ST10-6-15}$ and plTALE$_{ST10X-8-15}$) were used. Thus, plTALE$_{ST10Xs}$ are powerful effectors to increase the expression of the endogenous *FXN* gene in FRDA cells. These results demonstrate that the effector plTALE$_{TA}$ activates transcription initiation in the case of FRDA regardless of the number of GAA in intron 1. This is strongly suggestive that the development of a gene therapy with plTALE$_{TA}$ is, therefore, an effective strategy for treating this anomaly by targeting the main cause, namely, silencing the gene and decreasing the expression of frataxin.

We conducted *in vivo* experiments with the best plTALE$_{VP64}$ and plTALE$_{ST}$ in the YG8R mouse model of FRDA.[37,51] These effectors were delivered i.p. with AAV9s, which is the most frequently used vector for gene therapy.[35,36,52–54] These effectors were under a CAG promoter[55] permitting expression in most mouse tissues,[56,57] including the brain, heart, liver, and muscles, which are mainly involved in FRDA.[13] Following i.p. injection, the AAV9 distribution varied from organ to organ and was stronger in the heart, muscles, and liver than in the brain, confirming the previous results of our

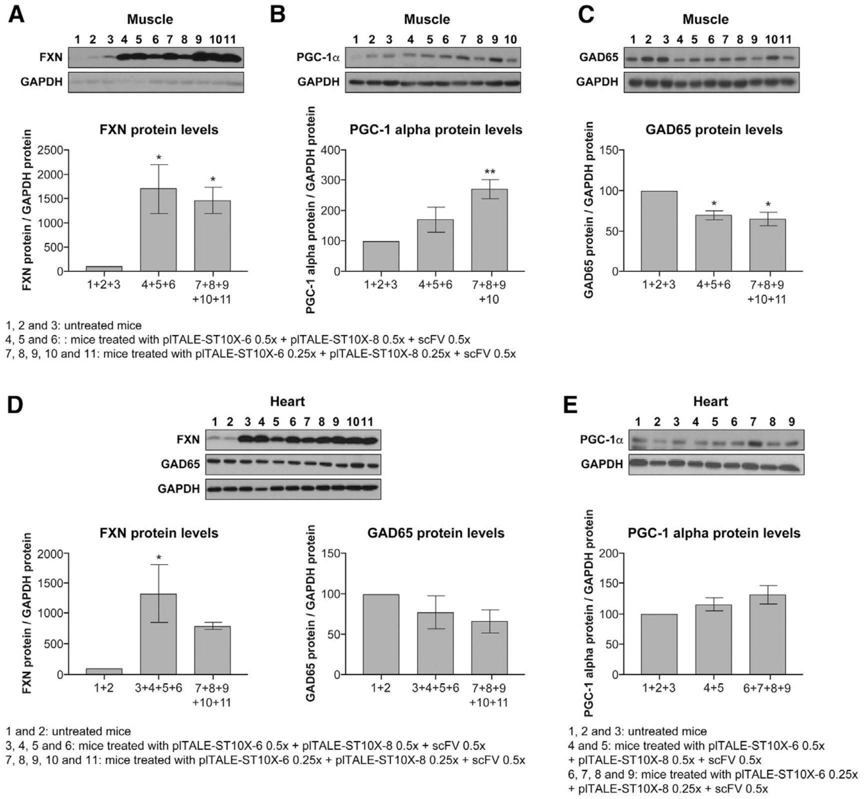

Figure 9. Expression Regulation of PGC-1 α and CAD65 Genes after Increased Expression of the Frataxin Protein in Muscles and Heart Treated Mice with AAV9-plTALE$_{ST10X}$

(A–C) The levels of expression of frataxin (A), PGC-1α (B), and GAD65 proteins (C) in the muscles of mice treated with AAV9-pTALE-ST10X-6 + AAV9- plTALE$_{ST10-8}$ + AAV9-scFv compared to untreated mice. (D and E) The levels of expression of frataxin and GAD65 (D) and PGC-1α proteins (E) in the heart of mice, which received the same treatment compared to untreated mice. *p < 0.05, **p < 0.003, ***p < 0.0003, and ****p < 0.0001. (A–E) Results are the average ± SEM.

group in 2014 and 2016.[34,58] A strong heart infection was also observed by Carroll et al.[59] and Mayra et al.[36] Although AAV9 is able to cross the blood-brain barrier, the delivery to the brain was low in our experiments.[60] The amount of virus in each organ proportionally influenced the expression of plTALE$_{VP64}$ and plTALE$_{ST}$. Thus, we observed a higher increase of *FXN* transcription by plTALE$_{ST-6}$ and plTALE$_{ST-8}$ in muscles and heart and no effect in the YG8R brain. Therefore, our results showed for the first time that TALE$_{ST}$ can increase the expression of an endogenous *FXN* gene *in vivo*.

The increase of the FRDA *FXN* gene expression *in vitro* and *in vivo* by plTALE$_{ST10X-6-15}$ and plTALE$_{ST10X-8-15}$ improved mitochondrial activity in FRDA cells as indicated by the increased aconitase activity and regulation of *PGC-1α* and *CAD65*.[42] Khonsari et al.[15] also observed a 2-fold increase of the aconitase activity in FRDA fibroblasts treated *in vitro* with lentiviruses expressing the frataxin protein.

Given our positive *in vivo* results, we now plan to test the plTALE effectors in the YG8sR mouse, a new FRDA model containing 300 GAA repeats.[38] Given that the AAV9 did not induce an increased *FXN* expression in the brain, there are new AAV serotypes such as AAV-PHP.eB, which have been recently described to better deliver genes to the brain;[61] we will test this new serotype. These tests will enable us to validate the *in vivo* effectiveness of the plTALEs to treat a FRDA mouse model before clinical trials.

Could the two plTALE$_{ST}$, which best increased frataxin expression, also increase the expression of other genes? To induce expression of an off-target gene, the plTALE$_{ST}$ would need to bind to an off-target sequence, which is in a promoter (not any genomic sequence as is the case for off-target mutations induced by TALE nucleases [TALENs] or by an active Cas9). Our experiments demonstrated that, even when some plTALE-VP64 or plTALE$_{ST}$ bind to a promoter region of a gene, they do not necessary induced gene expression. Indeed as illustrated in our Figure 1C, we have made several plTALE-VP64 targeting other sequences in the frataxin promoter (sequences 1, 2, 3, 11, F2, F3, and F4) and several plTALE$_{ST}$ targeting sequences (7, 10, and F4), which did NOT induce frataxin expression. Moreover, plTALE$_{ST}$ targeting shorter nucleotide sequences (i.e., 13 instead of 15 nt, sequences 6–13, 7–13, and 10–13 in Figure 3C) did NOT increase frataxin expression. Thus, the probability of inducing an off-target gene by our plTALE-ST10X-6-15 or plTALE-ST10X-8-15 is very low. However, for an eventual clinical application, studies of the off-target effects of the plTALEs using RNA sequencing (RNA-seq) and of the immune responses against the effectors may be required by the regulatory agencies.

In summary, our results showed that transcription activation by plTALE$_{ST}$ is a promising approach to treat FRDA by increasing the expression of the endogenous *FXN* gene. This approach may have several advantages over other therapeutic approaches. It may produce fewer off-target effects than genome editing with CRISPR or TALEN. This plTALE$_{VP64}$ and plTALE$_{ST}$ therapeutic approach may also be used for many other hereditary diseases due to haploinsufficiency.

MATERIALS AND METHODS

DNA Constructs

We used the Platinum TALEN kit (Kit 1000000043, Addgene, Cambridge, MA, USA)[28] to construct pCR3.1-plTALE$_{TAs}$. This kit contains 34 plasmids distributed as follows: 16 plasmids containing the various RVD modules and 8 plasmids containing a single RVD module and the FokI nuclease. In addition, the pFus2 plasmid permits

one to group 4 successive RVDs. The FokI nuclease was subsequently replaced by a transcriptional activator.

Platinum TALEs (plTALEs) are distinct from classical TALEs by two particularities. The first one is the variation of amino acids 4 and 30 of the RVD sequences in addition to variations of amino acids 12 and 13 found in conventional TALEs. The second particularity is that the RVDs are assembled using Golden Gate cloning steps with the BsaI enzyme in groups of 2 and 4 RVDs in a plasmid called pFus2 (Figure S1A). This technique is very effective compared to the assembly of modules containing 10 RVDs for the TALEN.[28,62] Three or four pFus2 plasmids each containing 2 or 4 RVDs are subsequently assembled using Golden Gate cloning steps, with the Esp3I enzyme in a single vector ptCMV-VR to obtain a plTALEN (plTALE nuclease) containing 13 or 15 RVDs (Figure S1B)[28] and a FokI nuclease. The FokI was subsequently replaced by a transcriptional activator (VP64, p300, or an ST) to construct a $plTALE_{VP64}$, $plTALE_{p300}$, or $plTALE_{ST}$ in the plasmid pCR3.1. This assembly technique used fewer starting plasmids (p1HD-P4HD, p1NG-p4NG, p1NI-p4NI, and p1NN-p4NN) compared to Golden Gate TALEN and TAL Effector Kit 2.0.

The $plTALE_{ST}$ induction system consists of a plTALE fused with an ST plasmid containing 10 or 24 epitopes (ST10X or ST24X).[32] The latter makes it possible to recruit several VP64 transcriptional activators fused with an scFv peptide (single-chain variable fragment), which is fused with the sfGFP-VP64-GB1 complex. The $plTALE_{ST10X}$ or $plTALE_{ST24X}$ proteins that target the sequences in the *FXN* gene promoter were expressed by a single pCR3.1-$plTALE_{ST}$ vector. The scFv-sfGFP-VP64-GB1 (scFv) complex was expressed by another vector pCR3.1-scFv-sfGFP-VP64-GB1.

The pAAV-$plTALE_{VP64}$, pAAV-$plTALE_{ST10Xs}$, and pAAV-scFv were first constructed in a pAAV_TALE-TF (VP64)-BB_V3 plasmid (Addgene, 42581, Cambridge, MA, USA). The latter contained a CAG promoter having a similar expression power as the CMV promoter.[26,28] It also contained an inverted terminal repeat (ITR) sequence at each end. The maximum insert size used in this viral vector was 4,700-bp different FRDA primary fibroblasts.

FRDA Fibroblasts

The $plTALE_{VP64s}$, $plTALE_{p300s}$, and $plTALE_{STs}$ were tested *in vitro* on patient FRDA primary fibroblasts GM04078 (FRDA4078) (obtained from the Coriell Institute, Camden, NJ, USA). These cells contain 541/420 GAA repeats. Subsequently, the best $plTALE_{VP64s}$ and $plTALE_{ST10Xs}$ were tested *in vitro* on FRDA primary fibroblasts of four different patients: FRDA66 (240/640), FRDA162 (355/805), FRDA4675 (255/1,140), and FRDA4743 (470/970) (generously provided by Dr. Napierala and Dr. Lynch, Children's Hospital of Philadelphia). The results of these tests were compared with the transcriptional activity of the *FXN* gene in fibroblasts of normal subjects: NED29178, NED36091, NED36320, and NED34769 obtained from the Coriell Institute.

The FRDA and NED cells were grown at 37°C under 5% CO_2 in DMEM (from Wisent, Saint-Jean-Baptiste, QC, Canada) with 10% fetal bovine serum (FBS) (Gibco, Burlington, ON, Canada), 1% antibiotics (penicillin and streptomycin, Gibco by Life Technologies, 15140-122) and 1% Non-Essential Amino Acid 100× Medium (NEAA, Wisent Bioproducts, 321-011-EL, Canada).

Nucleofection

One million FRDA cells were resuspended in 100 μL nucleofection solution (Amexa nucleofector, ESBE Scientific Products, St-Laurent, QC, Canada) and nucleofected with 10 μg plasmids using the program P022 (Figure 1F).[63] The cells were kept in culture for 2 or 3 days with a change of medium every 24 hr, and the expression of frataxin was analyzed by qRT-PCR and western blot.

Western Blot

After RNA extraction with Qiazol, the proteins were precipitated with isopropanol at room temperature (RT) and washed with 0.3 M guanidine hydrochloride and pure ethanol. A resuspension buffer (4% SDS, 0.025 M Tris HCl, 7.5% glycerol, 0.5% β-mercaptoethanol, and bromophenol blue) was used for protein denaturation at 100°C for 5 min. The concentration of proteins was quantified with the Amido black test.[64] Then 25–40 μg protein was separated on a 15% SDS-PAGE gel. Proteins were transferred onto a nitrocellulose membrane (Bio-Rad, Mississauga, ON, Canada).

After blocking, various primary and secondary antibodies were used as follows: anti-frataxin (ab110328, Abcam, Toronto, Canada), anti-GAPDH (Millipore Sigma MAB374, Etobicoke, ON, Canada), and anti-PGC-1α and anti-GAD65 (Thermo Fisher Scientific PA5-38022 and PA5-22260, Rockford, IL, USA). The secondary antibody used was a rabbit anti-mouse and anti-rabbit (Jackson ImmunoResearch, West Grove, PA, USA). After 3 washes in 0.1% PBS and 0.05% Tween for 10 min each, the membrane was revealed using the Clarity Western ECL Substrate kit (Bio-Rad, Mississauga, ON, Canada) and developed to visualize the protein bands. These were quantified with the ImageJ software.

Aconitase Test

Reversible modulation of aconitase was used as a biomarker of FRDA oxidative lesions. Aconitase activity was determined using a coupled enzymatic reaction in which the citrate is converted to isocitrate by the aconitase activity (Aconitase Activity Assay Kit Aconitase activity, Sigma-Aldrich MAK051, St. Louis, MI, USA). One million FRDA4078 cells treated with $plTALE_{ST10X-6-15}$ + with $plTALE_{ST10X-8-15}$ or 20–40 mg tissues from mice treated with AAV9-$plTALEs_{TA}$ were lysed in 120 μL test buffer solution. The insoluble material was removed by centrifugation at 800 × *g* for 10 min at 4°C. 10 μL aconitase activation solution was added to the cell extract followed by a 1- to 2-hr incubation on ice. 40 μL whole-cell lysates was added to the test reaction with and without enzyme mix plus 10 μL test buffer solution. The result of the aconitase activity is a colorimetric product, which was measured at 450 nm absorbance.

In Vivo Tests

Adeno-associated viruses serotype 9 (AAV9) were used to deliver the $plTALE_{TA}$ and induce the expression of the endogenous *FXN* gene *in vivo* in YG8R mice. The AAV9 vectors were produced by the Plateforme d'outils moléculaires, Centre de recherche CERVO (Québec, QC, Canada).

The best $plTALEs_{TA}$ were tested *in vivo* in the YG8R mouse model (The Jackson Laboratory, Sacramento, CA, USA) This transgenic mouse model contains two knockout mouse frataxin allele ($Fxn^{-/-}$) and two tandem copies of the human *FXN* derived from an FRDA patient containing, respectively, about 82 and 190 GAA trinucleotide sequence repeats.[37–39] The mice were reproduced in the CHUL animal facility, and all experiments were approved by the CRCHUL animal protection committee.

YG8R mice were injected i.p. around 7–11 days of age with 1.5, 3, 6, or 18×10^{11} virus particles. Their body weight was measured each week. They were sacrificed 1 month later, and tissues (Tibialis anterior muscles, heart, liver, and brain) were recovered, snap frozen in liquid nitrogen, and kept at −80°C until analysis.

RNA Extraction and Quantification of the Expression of *TALE*, *FXN*, and *GFP*

The fibroblast cells or the tissues were homogenized in Qiazol buffer (QIAGEN, Germantown, MD, USA), and total RNA was extracted using the RNeasy mini kit on-column DNase (QIAGEN, Hilden, DE, USA) treatment, following the manufacturer's instructions. Total RNA was measured using a NanoDrop ND-1000 Spectrophotometer (NanoDrop Technologies, Wilmington, DE, USA), and RNA quality was determined with the Agilent BioAnalyzer 2100 (Agilent Technologies, Santa Clara, CA, USA). First-strand cDNA synthesis was obtained using 3–4 μg isolated RNA in a reaction containing 200 U Superscript IV Rnase H-RT (Invitrogen Life Technologies, Burlington, ON, CA), 300 ng oligo-dT_{18}, 50 ng random hexamers, 50 mM Tris-HCl (pH 8.3), 75 mM KCl, 3 mM $MgCl_2$, 500 μM deoxynucleotide triphosphate, 5 mM dithiothreitol, and 40 U Protector RNase inhibitor (Roche Diagnostics, Indianapolis, IN, USA) in a final volume of 50 μL. The mixture was incubated at 25°C for 10 min and at 50°C for 20 min and inactivated at 80°C for 10 min. A PCR purification kit (QIAGEN, Hilden, DE, USA) was used to purify the cDNA. The cDNA corresponding to 20 ng total RNA was used to perform fluorescent-based real-time PCR quantification using the LightCycler 480 (Roche Diagnostics, Mannheim, DE, USA) and normalized with the expression of GAPDH and/or HPRT1. The qRT-PCR analyses were done by the Plateforme d'Expression Génique of the Centre Génomique du Centre de recherche du CHU de Québec (CRCHUL). The different primers used are listed in Table S1.

DNA Extraction from Tissues

DNA was extracted from different tissues (muscle, liver, heart, and brain). Briefly, a part of the tissue was recovered incubated with 50 μL proteinase K (10 mg/mL) in a lysis buffer at 56°C until the solution became clear. Digested tissues were then mixed with 500 μL solution of phenol/chloroform/isoamyl alcohol (25:24:1; BioShop Canada) and centrifuged 3 min at 13,000 rpm. The upper solution was recovered and mixed with the same volume of chloroform and centrifuged again. The upper solution was recovered and 50 μL of 5 M sodium chloride was added before the addition of 1 mL 100% ethanol. After centrifugation for 8 min at 12,000 rpm, the pellets were washed in 70% alcohol before another centrifugation. Pellets were dried before DNA suspension in sterile water. A qPCR was made to detect v.p. in the tissues.

Frataxin Protein Quantification with the Dipstick Assay Kit

The protein concentration was estimated using the PierceTM BCA Protein Assay Kit (Thermo Scientific, Waltham, MA, USA). The human frataxin protein was quantified using the Dipstick Array (ab109881, Abcam, Cambridge, MA, USA). A standard curve was also done at the same time with recombinant human frataxin protein ranging from 0.075 to 1.2 ng of frataxin (110353, Abcam, Toronto, ON, Canada) to quantify the frataxin protein in the tissues in nanogram/microgram total protein.

Microscopy

For light microscopy, part of the tissue was incubated with 30% sucrose overnight before including it in Tissue-Tek O.C.T. Compound (Sakura Finetek, Torrance, CA, USA) for freezing in liquid nitrogen. Sections of 12 μm were cut on a Leica CM3050S (Leica Biosystems, Concorde, ON, Canada) and stored at −20°C. The slides were fixed with 10% neutral buffered formalin (Fisher Scientific, Ottawa, ON, Canada) for 10 min before 3 washes of 5 min in PBS. In the second wash, Hoechst 33258 dye (Sigma-Aldrich Canada, Oakville, ON, Canada) was incorporated at 0.2 ng/μL. The slides were mounted with PBS/glycerol and observed by confocal microscopy Leica DMI6000B (GFP: excitation 491 nm, emission 536/40 nm; Dapi Widefield: excitation 350/50, dichroic 400LP, emission 456/50) with an objective 63× in glycerol immersion (HCX PL APO63X/1, 30Glyc).

Statistical Analyses

The various tests and treatments were carried out several times, with n varying between 2 and 10 times. The results were presented as average ± SEM. Statistical analysis was performed using one-way ANOVA with GraphPad Prism7 software (GraphPad, LaJolla, CA, USA). The p values are indicated in each figure. Statistics: one-way ANOVA was used at 0.05 (95% confidence interval); *$p < 0.05$, **$p < 0.003$, ***$p < 0.0003$, and ****$p < 0.0001$.

SUPPLEMENTAL INFORMATION

Supplemental Information includes Supplemental Results, eight figures, and one table and can be found with this article online at https://doi.org/10.1016/j.omtn.2018.04.009.

AUTHOR CONTRIBUTIONS

K.C., J.R., D.L.O., and P.C. conducted the *in vitro* experiments. C.G., K.C., and D.L.O. conducted the *in vivo* experiments. K.C., C.G., D.L.O., and J.P.T. contributed to the writing of the article.

CONFLICTS OF INTEREST

The authors declare no conflicts of interest.

ACKNOWLEDGMENTS

This work was supported by a research contract from AmorChem Venture Fund Inc. (Montréal, Canada) and grants from Ataxia Canada and the ThéCel FRQS network. We would like to thank Nathalie Paquet, Nizar Chetoui, Caroline Genest, and Chantale Maltais for their availability and flexibility. We also want to thank Véronique Dorval, our dear colleague who passed away last year.

REFERENCES

1. Gomes, C.M., and Santos, R. (2013). Neurodegeneration in Friedreich's ataxia: from defective frataxin to oxidative stress. Oxid. Med. Cell. Longev. *2013*, 487534.
2. Pandolfo, M. (2008). Friedreich ataxia. Arch. Neurol. *65*, 1296–1303.
3. Delatycki, M.B., Williamson, R., and Forrest, S.M. (2000). Friedreich ataxia: an overview. J. Med. Genet. *37*, 1–8.
4. Chutake, Y.K., Costello, W.N., Lam, C.C., Parikh, A.C., Hughes, T.T., Michalopulos, M.G., Pook, M.A., and Bidichandani, S.I. (2015). FXN Promoter Silencing in the Humanized Mouse Model of Friedreich Ataxia. PLoS ONE *10*, e0138437.
5. Potdar, P.D., and Raghu, A. (2013). Review on Molecular Diagnostic Techniques in Friedreich's Ataxia. Annu. Rev. Res. Biol. *3*, 659–677.
6. Wells, R.D. (2008). DNA triplexes and Friedreich ataxia. FASEB J. *22*, 1625–1634.
7. De Biase, I., Chutake, Y.K., Rindler, P.M., and Bidichandani, S.I. (2009). Epigenetic silencing in Friedreich ataxia is associated with depletion of CTCF (CCCTC-binding factor) and antisense transcription. PLoS ONE *4*, e7914.
8. Bürk, K. (2017). Friedreich Ataxia: current status and future prospects. Cerebellum Ataxias *4*, 4.
9. Kumari, D., Biacsi, R.E., and Usdin, K. (2011). Repeat expansion affects both transcription initiation and elongation in friedreich ataxia cells. J. Biol. Chem. *286*, 4209–4215.
10. Chutake, Y.K., Costello, W.N., Lam, C., and Bidichandani, S.I. (2014). Altered nucleosome positioning at the transcription start site and deficient transcriptional initiation in Friedreich ataxia. J. Biol. Chem. *289*, 15194–15202.
11. Campuzano, V., Montermini, L., Lutz, Y., Cova, L., Hindelang, C., Jiralerspong, S., Trottier, Y., Kish, S.J., Faucheux, B., Trouillas, P., et al. (1997). Frataxin is reduced in Friedreich ataxia patients and is associated with mitochondrial membranes. Hum. Mol. Genet. *6*, 1771–1780.
12. Al-Mahdawi, S., Sandi, C., Mouro Pinto, R., and Pook, M.A. (2013). Friedreich ataxia patient tissues exhibit increased 5-hydroxymethylcytosine modification and decreased CTCF binding at the FXN locus. PLoS ONE *8*, e74956.
13. Koeppen, A.H. (2011). Friedreich's ataxia: pathology, pathogenesis, and molecular genetics. J. Neurol. Sci. *303*, 1–12.
14. Bulteau, A.-L., O'Neill, H.A., Kennedy, M.C., Ikeda-Saito, M., Isaya, G., and Szweda, L.I. (2004). Frataxin acts as an iron chaperone protein to modulate mitochondrial aconitase activity. Science *305*, 242–245.
15. Khonsari, H., Schneider, M., Al-Mahdawi, S., Chianea, Y.G., Themis, M., Parris, C., Pook, M.A., and Themis, M. (2016). Lentivirus-meditated frataxin gene delivery reverses genome instability in Friedreich ataxia patient and mouse model fibroblasts. Gene Ther. *23*, 846–856.
16. Shinnick, J.E., Isaacs, C.J., Vivaldi, S., Schadt, K., and Lynch, D.R. (2016). Friedreich Ataxia and nephrotic syndrome: a series of two patients. BMC Neurol. *16*, 3.
17. Carletti, B., and Piemonte, F. (2014). Friedreich's Ataxia: A Neuronal Point of View on the Oxidative Stress Hypothesis. Antioxidants *3*, 592–603.
18. Lynch, D.R., Farmer, J.M., and Wilson, R.B. (2007). Mortality in Friedreich's Ataxia. Tex. Heart Inst. J. *34*, 502–503, author reply 503–504.
19. Rustin, P., von Kleist-Retzow, J.-C., Chantrel-Groussard, K., Sidi, D., Munnich, A., and Rötig, A. (1999). Effect of idebenone on cardiomyopathy in Friedreich's ataxia: a preliminary study. Lancet *354*, 477–479.
20. Parkinson, M.H., Schulz, J.B., and Giunti, P. (2013). Co-enzyme Q10 and idebenone use in Friedreich's ataxia. J. Neurochem. *126* (*Suppl 1*), 125–141.
21. Vyas, P.M., Tomamichel, W.J., Pride, P.M., Babbey, C.M., Wang, Q., Mercier, J., Martin, E.M., and Payne, R.M. (2012). A TAT-frataxin fusion protein increases lifespan and cardiac function in a conditional Friedreich's ataxia mouse model. Hum. Mol. Genet. *21*, 1230–1247.
22. Choy, B., and Green, M.R. (1993). Eukaryotic activators function during multiple steps of preinitiation complex assembly. Nature *366*, 531–536.
23. Memedula, S., and Belmont, A.S. (2003). Sequential recruitment of HAT and SWI/SNF components to condensed chromatin by VP16. Curr. Biol. *13*, 241–246.
24. Hilton, I.B., D'Ippolito, A.M., Vockley, C.M., Thakore, P.I., Crawford, G.E., Reddy, T.E., and Gersbach, C.A. (2015). Epigenome editing by a CRISPR-Cas9-based acetyltransferase activates genes from promoters and enhancers. Nat. Biotechnol. *33*, 510–517.
25. Hall, D.B., and Struhl, K. (2002). The VP16 activation domain interacts with multiple transcriptional components as determined by protein-protein cross-linking in vivo. J. Biol. Chem. *277*, 46043–46050.
26. Moore, R., Chandrahas, A., and Bleris, L. (2014). Transcription activator-like effectors: a toolkit for synthetic biology. ACS Synth. Biol. *3*, 708–716.
27. Sanjana, N.E., Cong, L., Zhou, Y., Cunniff, M.M., Feng, G., and Zhang, F. (2012). A transcription activator-like effector toolbox for genome engineering. Nat. Protoc. *7*, 171–192.
28. Sakuma, T., Ochiai, H., Kaneko, T., Mashimo, T., Tokumasu, D., Sakane, Y., Suzuki, K., Miyamoto, T., Sakamoto, N., Matsuura, S., and Yamamoto, T. (2013). Repeating pattern of non-RVD variations in DNA-binding modules enhances TALEN activity. Sci. Rep. *3*, 3379.
29. Sakuma, T., Hosoi, S., Woltjen, K., Suzuki, K., Kashiwagi, K., Wada, H., Ochiai, H., Miyamoto, T., Kawai, N., Sasakura, Y., et al. (2013). Efficient TALEN construction and evaluation methods for human cell and animal applications. Genes Cells *18*, 315–326.
30. Wang, X., Wang, P., Fu, Z., Ji, H., Qu, X., Zeng, H., Zhu, X., Deng, J., Lu, P., Zha, S., et al. (2015). Designed transcription activator-like effector proteins efficiently induced the expression of latent HIV-1 in latently infected cells. AIDS Res. Hum. Retroviruses *31*, 98–106.
31. Hu, J., Lei, Y., Wong, W.-K., Liu, S., Lee, K.-C., He, X., You, W., Zhou, R., Guo, J.T., Chen, X., et al. (2014). Direct activation of human and mouse Oct4 genes using engineered TALE and Cas9 transcription factors. Nucleic Acids Res. *42*, 4375–4390.
32. Tanenbaum, M.E., Gilbert, L.A., Qi, L.S., Weissman, J.S., and Vale, R.D. (2014). A protein-tagging system for signal amplification in gene expression and fluorescence imaging. Cell *159*, 635–646.
33. Rinaldi, F.C., Doyle, L.A., Stoddard, B.L., and Bogdanove, A.J. (2017). The effect of increasing numbers of repeats on TAL effector DNA binding specificity. Nucleic Acids Res. *45*, 6960–6970.
34. Gérard, C., Xiao, X., Filali, M., Coulombe, Z., Arsenault, M., Couet, J., Li, J., Drolet, M.C., Chapdelaine, P., Chikh, A., and Tremblay, J.P. (2014). An AAV9 coding for frataxin clearly improved the symptoms and prolonged the life of Friedreich ataxia mouse models. Mol. Ther. Methods Clin. Dev. *1*, 14044.
35. Machida, A., Kuwahara, H., Mayra, A., Kubodera, T., Hirai, T., Sunaga, F., Tajiri, M., Hirai, Y., Shimada, T., Mizusawa, H., and Yokota, T. (2013). Intraperitoneal administration of AAV9-shRNA inhibits target gene expression in the dorsal root ganglia of neonatal mice. Mol. Pain *9*, 36.
36. Mayra, A., Tomimitsu, H., Kubodera, T., Kobayashi, M., Piao, W., Sunaga, F., Hirai, Y., Shimada, T., Mizusawa, H., and Yokota, T. (2011). Intraperitoneal AAV9-shRNA inhibits target expression in neonatal skeletal and cardiac muscles. Biochem. Biophys. Res. Commun. *405*, 204–209.
37. Perdomini, M., Hick, A., Puccio, H., and Pook, M.A. (2013). Animal and cellular models of Friedreich ataxia. J. Neurochem. *126* (*Suppl 1*), 65–79.
38. Anjomani Virmouni, S., Ezzatizadeh, V., Sandi, C., Sandi, M., Al-Mahdawi, S., Chutake, Y., and Pook, M.A. (2015). A novel GAA-repeat-expansion-based mouse model of Friedreich's ataxia. Dis. Model. Mech. *8*, 225–235.
39. Sandi, C., Sandi, M., Jassal, H., Ezzatizadeh, V., Anjomani-Virmouni, S., Al-Mahdawi, S., and Pook, M.A. (2014). Generation and characterisation of

Friedreich ataxia YG8R mouse fibroblast and neural stem cell models. PLoS ONE *9*, e89488.

40. Rufini, A., Cavallo, F., Condò, I., Fortuni, S., De Martino, G., Incani, O., Di Venere, A., Benini, M., Massaro, D.S., Arcuri, G., et al. (2015). Highly specific ubiquitin-competing molecules effectively promote frataxin accumulation and partially rescue the aconitase defect in Friedreich ataxia cells. Neurobiol. Dis. *75*, 91–99.
41. Condò, I., Malisan, F., Guccini, I., Serio, D., Rufini, A., and Testi, R. (2010). Molecular control of the cytosolic aconitase/IRP1 switch by extramitochondrial frataxin. Hum. Mol. Genet. *19*, 1221–1229.
42. Lin, H., Magrane, J., Rattelle, A., Stepanova, A., Galkin, A., Clark, E.M., Dong, Y.N., Halawani, S.M., and Lynch, D.R. (2017). Early cerebellar deficits in mitochondrial biogenesis and respiratory chain complexes in the KIKO mouse model of Friedreich ataxia. Dis. Model. Mech. *10*, 1343–1352.
43. Silva, A.M., Brown, J.M., Buckle, V.J., Wade-Martins, R., and Lufino, M.M.P. (2015). Expanded GAA repeats impair FXN gene expression and reposition the FXN locus to the nuclear lamina in single cells. Hum. Mol. Genet. *24*, 3457–3471.
44. Ghosh, A.K., and Varga, J. (2007). The transcriptional coactivator and acetyltransferase p300 in fibroblast biology and fibrosis. J. Cell. Physiol. *213*, 663–671.
45. Li, K., Singh, A., Crooks, D.R., Dai, X., Cong, Z., Pan, L., Ha, D., and Rouault, T.A. (2010). Expression of human frataxin is regulated by transcription factors SRF and TFAP2. PLoS ONE *5*, e12286.
46. Chapdelaine, P., Coulombe, Z., Chikh, A., Gérard, C., and Tremblay, J.P. (2013). A Potential New Therapeutic Approach for Friedreich Ataxia: Induction of Frataxin Expression With TALE Proteins. Mol. Ther. Nucleic Acids *2*, e119.
47. Anthony, K., More, A., and Zhang, X. (2014). Activation of silenced cytokine gene promoters by the synergistic effect of TBP-TALE and VP64-TALE activators. PLoS ONE *9*, e95790.
48. Xu, X., Tao, Y., Gao, X., Zhang, L., Li, X., Zou, W., Ruan, K., Wang, F., Xu, G.L., and Hu, R. (2016). A CRISPR-based approach for targeted DNA demethylation. Cell Discov. *2*, 16009.
49. Ji, H., Jiang, Z., Lu, P., Ma, L., Li, C., Pan, H., Fu, Z., Qu, X., Wang, P., Deng, J., et al. (2016). Specific Reactivation of Latent HIV-1 by dCas9-SunTag-VP64-mediated Guide RNA Targeting the HIV-1 Promoter. Mol. Ther. *24*, 508–521.
50. Mahfouz, M.M., and Li, L. (2011). TALE nucleases and next generation GM crops. GM Crops *2*, 99–103.
51. Martelli, A., Napierala, M., and Puccio, H. (2012). Understanding the genetic and molecular pathogenesis of Friedreich's ataxia through animal and cellular models. Dis. Model. Mech. *5*, 165–176.
52. Aschauer, D.F., Kreuz, S., and Rumpel, S. (2013). Analysis of transduction efficiency, tropism and axonal transport of AAV serotypes 1, 2, 5, 6, 8 and 9 in the mouse brain. PLoS ONE *8*, e76310.
53. Yu, W., Mookherjee, S., Chaitankar, V., Hiriyanna, S., Kim, J.-W., Brooks, M., Ataeijannati, Y., Sun, X., Dong, L., Li, T., et al. (2017). Nrl knockdown by AAV-delivered CRISPR/Cas9 prevents retinal degeneration in mice. Nat. Commun. *8*, 14716.
54. Daya, S., and Berns, K.I. (2008). Gene therapy using adeno-associated virus vectors. Clin. Microbiol. Rev. *21*, 583–593.
55. Alexopoulou, A.N., Couchman, J.R., and Whiteford, J.R. (2008). The CMV early enhancer/chicken β actin (CAG) promoter can be used to drive transgene expression during the differentiation of murine embryonic stem cells into vascular progenitors. BMC Cell Biol. *9*, 2.
56. Bell, C.L., Vandenberghe, L.H., Bell, P., Limberis, M.P., Gao, G.-P., Van Vliet, K., Agbandje-McKenna, M., and Wilson, J.M. (2011). The AAV9 receptor and its modification to improve in vivo lung gene transfer in mice. J. Clin. Invest. *121*, 2427–2435.
57. Inagaki, K., Fuess, S., Storm, T.A., Gibson, G.A., McTiernan, C.F., Kay, M.A., and Nakai, H. (2006). Robust systemic transduction with AAV9 vectors in mice: efficient global cardiac gene transfer superior to that of AAV8. Mol. Ther. *14*, 45–53.
58. Chapdelaine, P., Gérard, C., Sanchez, N., Cherif, K., Rousseau, J., Ouellet, D.L., Jauvin, D., and Tremblay, J.P. (2016). Development of an AAV9 coding for a 3XFLAG-TALEfrat#8-VP64 able to increase in vivo the human frataxin in YG8R mice. Gene Ther. *23*, 606–614.
59. Carroll, K.J., Makarewich, C.A., McAnally, J., Anderson, D.M., Zentilin, L., Liu, N., Giacca, M., Bassel-Duby, R., and Olson, E.N. (2016). A mouse model for adult cardiac-specific gene deletion with CRISPR/Cas9. Proc. Natl. Acad. Sci. USA *113*, 338–343.
60. Manfredsson, F.P., Rising, A.C., and Mandel, R.J. (2009). AAV9: a potential blood-brain barrier buster. Mol. Ther. *17*, 403–405.
61. Chan, K.Y., Jang, M.J., Yoo, B.B., Greenbaum, A., Ravi, N., Wu, W.-L., Sánchez-Guardado, L., Lois, C., Mazmanian, S.K., Deverman, B.E., and Gradinaru, V. (2017). Engineered AAVs for efficient noninvasive gene delivery to the central and peripheral nervous systems. Nat. Neurosci. *20*, 1172–1179.
62. Cermak, T., Doyle, E.L., Christian, M., Wang, L., Zhang, Y., Schmidt, C., Baller, J.A., Somia, N.V., Bogdanove, A.J., and Voytas, D.F. (2011). Efficient design and assembly of custom TALEN and other TAL effector-based constructs for DNA targeting. Nucleic Acids Res. *39*, e82.
63. Zeitelhofer, M., Vessey, J.P., Xie, Y., Tübing, F., Thomas, S., Kiebler, M., and Dahm, R. (2007). High-efficiency transfection of mammalian neurons via nucleofection. Nat. Protoc. *2*, 1692–1704.
64. Chapdelaine, P., Vignola, K., and Fortier, M.A. (2001). Protein estimation directly from SDS-PAGE loading buffer for standardization of samples from cell lysates or tissue homogenates before Western blot analysis. Biotechniques *31*, 478–482.

OMTN, Volume *12*

Supplemental Information

Increased Frataxin Expression Induced in Friedreich Ataxia Cells by Platinum TALE-VP64s or Platinum TALE-SunTag

Khadija Cherif, Catherine Gérard, Joël Rousseau, Dominique L. Ouellet, Pierre Chapdelaine, and Jacques P. Tremblay

Increased frataxin expression induced in Friedreich ataxia cells

by platinum TALE-VP64s or platinum TALE-SunTag

Khadija Cherif, Catherine Gérard, Joël Rousseau, Dominique L. Ouellet,

Pierre Chapdelaine and Jacques P. Tremblay*

Centre de Recherche du CHU de Québec-Université Laval and Département

de Médecine Moléculaire de l'Université Laval Québec, Canada.

Supplementary results

Assembly of the various TALEN

The assembled TALENs were verified by PCR (Figure 1S).

Lack of improvement in the specificity and affinity of plTALEs with new nRVD sequences (nplTALEs)

To further improve our therapeutic approach, we constructed plTALEs with new RVDs (nRVDs) [1] (i.e., nplTALE$_{VP64s}$) to increase the specificity and affinity of RVDs. First, the mutations of the nucleotides encoding the amino acid at position 12 coding for the RVD were made in the plasmids pCR3.1-plTALE$_{VP64-8-15}$ and pCR3.1-plTALE-$_{VP64-F4-15}$. These mutations replaced the RVDs NI, NG, and NN by CI, HG and EN respectively to generate the new RVDs. These new RVDs also contained 34 amino acids and were different from normal RVDs only by substitutions of the nucleotides encoding the amino acid in position 12. These base changes were AAC to TGC (to modify the asparagine into a cysteine, thus changing the RVDs NI into CI), AAC to CAC (to modify the asparagine into a histidine, thus changing the RVD NG into HG) and AAC to GAG (to modify the asparagine into a glutamic acid, thus changing the RVD NN into EN) (Figure S2A).

Thereafter, 2 new nplTALE$_{VP64s}$ with 15 nRVDs were built according to the same protocol that was used to build the plTALE$_{VP64s}$. These nplTALE$_{VP64s}$ targeted 2 sequences of 15 nucleotides sequences (i.e., *FXN* sequences 8 and F4) (Figure S2B). These 2 nplTALE$_{VP64s}$ were nucleofected in FRDA4078 cells to analyze 3 days later their effects on the transcriptional activity of the *FXN* gene. QRT-PCR analyzes (or analysis) were performed to quantify the number of *FXN* mRNA

copies. The transcriptional activity was normalized to GAPDH and HPRT, and compared to negative controls, i.e., FRDA4078 cells untreated or treated with an empty pCR3.1 plasmid (Figure S2C). The results showed that *FXN* mRNAs was not increased in the cells treated with nplTALE$_{VP64-8-15}$ and nplTALE$_{VP64-F4-15}$ compared to the negative controls. The *FXN* mRNA normalized with GAPDH and HPRT mRNAs did not change relative to the negative controls. Thus these 2 nplTALE$_{VP64s}$ with nRVDs (i.e., CI, NN and NG) did not activate the *FXN* gene in FRDA4078 cells. The efficacy of these 2 nplTALE$_{VP64s}$ was decreased compared to plTALEs$_{VP64s}$ targeting the same sequences.

Epigenetic change of the *FXN* gene with p300 acetyltransferase fused with plTALEs

P300 is a histone acetyltransferase (HAT) that regulates the transcription and remodeling of chromatin by histone acetylation. We fused p300 with plTALEs targeting either the transcription initiation region or a sequence in intron 1 of the *FXN* gene. These plTALE$_{p300}$ have been reported to be more effective to induce gene transcription after histone H3-lysine acetylation [2]. We constructed 3 plTALE$_{p300s}$ (Figure S2D) targeting 3 sequences (i.e., 6, 8 and F4) of the *FXN* gene. FRDA4087 cells were nucleofected with each of these 3 plTALE$_{p300s}$. The *FXN* gene transcription of these treated cells was compared 3 days later with the negative controls (untreated cells and cells treated with empty pCR3.1) (Figure S2E). When the *FXN* mRNA was normalized with either GAPDH or HPRT, the increases obtained with plTALE$_{p300-6-15}$ and with plTALE$_{p300-8-15}$ reached significant levels, but their effects were not as much as the effect of plTALE$_{VP64.}$

The effects of plTALE$_{p300s}$ were very low compared to those of plTALE$_{VP64s}$ targeting the same sequences. Thus, to activate the endogenous *FXN* gene, VP64 effectors are more potent than p300 effectors fused with plTALEs targeting sequences either in the promoter or in intron 1 of the *FXN* gene.

Study of the synergistic effects of plTALE$_{VP64s}$ and plTALE$_{p300}$ on *FXN* gene transcription in FRDA4078 cells

To study the synergistic effect of several plTALE$_{VP64}$ together, we treated FRDA4078 with the combinations of the 2, 3 or 4 most effective plTALE$_{VP64s}$. The *FXN* mRNAs increased even more when we treated these cells with several plTALE$_{VP64s}$ compared to the negative controls. The synergistic effect increased the *FXN* transcription normalized with GAPDH up to 3 folds when 4 effectors targeting sequences 6, 8, 10 and F4 were used (Figure 2A and B).

Analysis of the expression of the frataxin protein in cells treated by the different combinations of plTALE$_{VP64s}$ and in the negative control showed an increase of slightly more than 2.5 folds with the combination of plTALE$_{6\text{-}15}$ and plTALE$_{8\text{-}15}$. The combination of 3 plTALEs only increased frataxin expression by 1.9 fold over the negative control (Figure 2D and E). Therefore, these 3 plTALEs do not have a synergistic effect on the expression of frataxin compared with the effect of a single plTALE (Figure 2D and E).

The synergistic effects of the various combinations of plTALE$_{VP64s}$ with plTALE$_{p300s}$ and several plTALE$_{p300s}$ together, targeting the different sequences of the *FXN* gene, were investigated in FRDA4078 cells (Figure S3A and B). The transcriptional activity of the *FXN* gene was increased about 2 folds by two combinations: plTALE$_{VP64\text{-}6\text{-}15}$ with plTALE$_{p300\text{-}8\text{-}15}$ and plTALE$_{VP64\text{-}8\text{-}15}$ with plTALE$_{p300\text{-}6\text{-}15}$. Our previous results had shown that plTALE$_{VP64\text{-}6\text{-}15}$ or plTALE$_{VP64\text{-}8\text{-}15}$ alone also increased the transcriptional activity of the *FXN* gene in the FRDA4078 cells by only 2 folds. Thus, the effect of the combination of these 2 plTALEs (VP64 + p300) did not activate the *FXN* transcription more than a plTALE$_{VP64}$ alone. However, the combination of plTALE$_{p300\text{-}6\text{-}15}$ with plTALE$_{p300\text{-}8\text{-}15}$ significantly increased *FXN* transcription by 2 folds compared to the controls showing a small synergistic effect. This activity was equivalent to the effect of a single plTALE$_{VP64}$ with 15 RVD targeting sequences 6, 8 or F4.

Labelling the target locus of gene frataxin with plTALE$_{ST10X}$ expressed with the CAG or CMV promoter

We subsequently constructed pAAV-plTALE$_{ST10X-6-15}$ and pAAV-plTALE$_{ST10X-8-15}$. We also made the pAAV-scFv (pAAV-scFv-sfGFP-VP64-GB1-NLS). These pAAV plasmids contained a CAG promoter while our previous pCR3.1 plasmid contained a CMV promoter. The new constructions were initially tested *in vitro* in FRDA4078 cells using the same nucleofection protocol to compare their effectiveness relative to the previous pCR3.1 plasmids. The plTALE$_{ST10X}$ effectors expressed by pAAV and pCR3.1 similarly increased *FXN* transcriptional activity (Figure S4A and B). *FXN* transcription was increased by more than 8 folds by plTALE$_{ST10X-6-15}$ expressed either by pCR3.1 or by pAAV. This activity was also increased about 5 folds by plTALE$_{ST10X-8-15}$ expressed by pAAV and by about 8 folds by plTALE$_{ST10X-8-15}$ expressed by pCR3.1. These two effectors expressed either by pCR3.1 or pAAV synergistically increased the *FXN* transcription more than 18 folds.

Microscopic observation of the nuclei of the cells treated with this ST system revealed a much brighter sfGFP fluorescence in the nuclei of the cells nucleofected with a combination plTALE$_{ST10X-6-15}$ and plTALE$_{ST10X-8-15}$, which recruited a total of 20 scFv-sfGFP-VP64s (i.e., 10 tags for each plTALE) compared with the negative controls treated with a plasmid, which did not express the plTALE$_{ST10X}$, and a plasmid, which expressed only scFv-sfGFP-VP64 (Figure S4C). The sfGFP of the latter was expressed in the cytoplasm and in the nucleus but did not labelled the nuclei intensively in the absence of plTALE$_{ST}$. With the SunTag system, the two plTALE$_{ST10X}$ (targeting sequences 6 and 8 separated by 10 nucleotides) recruited of 20 scFv-sfGFP-VP64 leading to an intense fluorescent labeling of the nuclei confirming the *FXN* targeting by the 2 plTALE$_{ST10X}$.

In vitro treatment of FRDA4078 cells with pAAV-plTALE$_{ST10Xs}$ or pCR3.1-plTALE$_{ST10Xs}$ activated the expression of the endogenous *FXN* gene. However, microscopic observations of the cells treated with these effectors showed a much brighter nuclear labeling of cells treated with

pCR3.1 plasmids than with pAAV plasmids (Figure S4D). The signal difference may be due to the different promoters.

Bibliography

1. Miller, JC, Zhang, L, Xia, DF, Campo, JJ, Ankoudinova, IV, Guschin, DY, *et al.* (2015). Improved specificity of TALE-based genome editing using an expanded RVD repertoire. *Nature Methods* **12**: 465.
2. Hilton, IB, D'Ippolito, AM, Vockley, CM, Thakore, PI, Crawford, GE, Reddy, TE, *et al.* (2015). Epigenome editing by a CRISPR/Cas9-based acetyltransferase activates genes from promoters and enhancers. *Nature biotechnology* **33**: 510-517.

Figures and legends

Figure S1

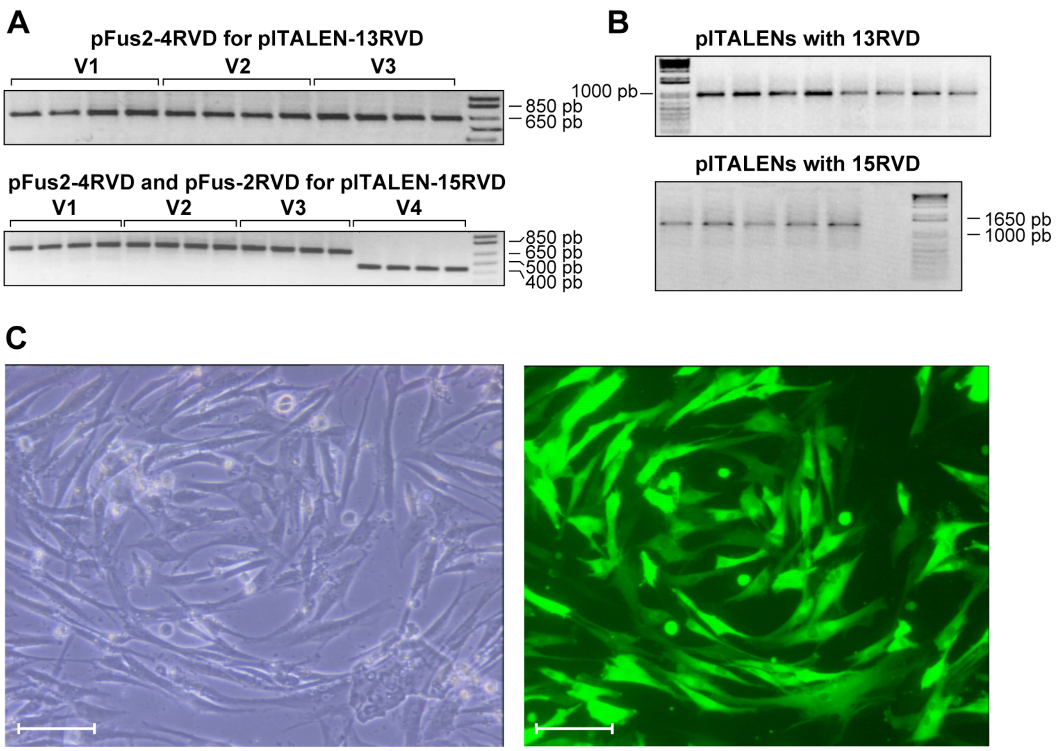

Figure S1: Validation of the construction of plTALE$_{VP64s}$ targeting different sequences of the *FXN* gene and nucleofected in FRDA4078 fibroblasts.

A) The figure illustrates the PCR amplification of sequences containing 4 RVDs (V1, V2 and V3) corresponding to the first 12 RVDs of plTALEN-13RVD or plTALEN-15RVD. The V4 is the PCR amplification of the assembly of 2 RVDs corresponding to the thirteenth and fourteenth RVDs of plTALEN-15RVD. **B)** PCR to select the adequate final assembly of plTALENs with 13 RVDs (1332 pb) and 15 RVDs (1532 pb). **C)** Representative image of FRDA4078 fibroblasts (left side in phase contrast and right side in fluorescence), 24 h after nucleofection with a plasmid containing the GFP gene. Scale bars in C are 100 μm.

Figure S2

A

Target Nucleotide	Normal RVD	New RVD
A	**NI**<br>**AA**C ATA	**CI**<br>**TG**C ATA
T	**NG**<br>**A**AC GGC	**HG**<br>**C**AC GGC
C	**HD**<br>CAC GAT	**HD**<br>CAC GAT
G	**NN**<br>**AAC** AAC	**EN**<br>**GAG** AAC

B

npITALE-VP64	Target sequence	RVD sequence
**npITALE-VP64-8-15**	TCCTGAGGTCTAACCT	HD HD HG EN CI EN EN HG HD HG CI CI HD HD HG
**npITALE-VP64-F4-15**	TGCGCAGGGAGGCGCC	EN HD EN HD CI EN EN EN CI EN EN HD EN HD HD

C

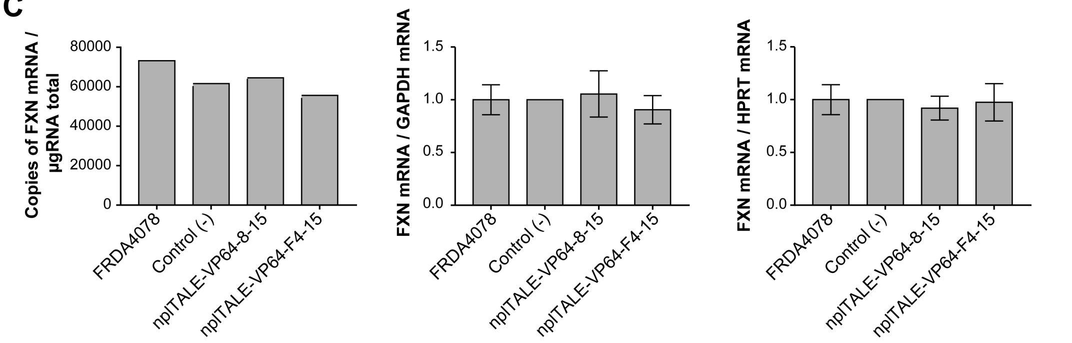

D

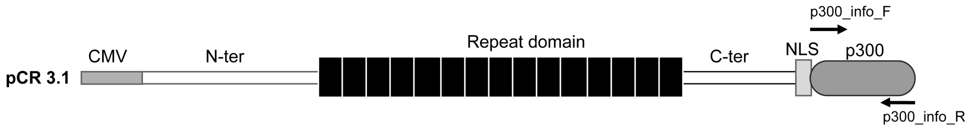

E

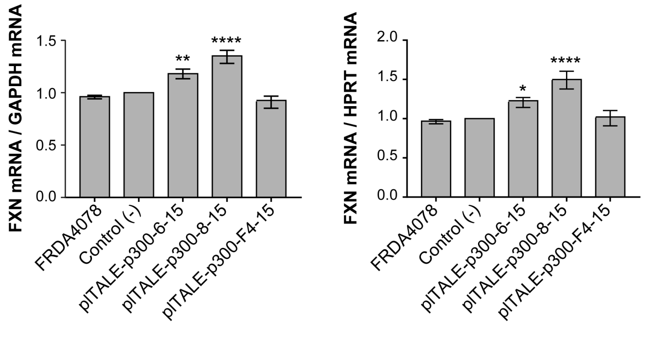

Figure S2: Study of the effects of the npITALE$_{VP64s}$ and pITALE$_{p300s}$.

A) The amino acid pairs and the codons of the normal and new RVDs targeting a specific nucleotide. **B)** List of npITALE$_{VP64s}$ with their new RVD sequences and their target sequences. **C)** *FXN* gene mRNA in control cells and in FRDA4078 cells treated with npITALE$_{VP64}$. **D)** Scheme

of plTALE$_{P300}$ in pCR3.1 containing the CMV promoter, N-ter, repeat domain, C-ter, NLS and p300. **E)** *FXN* gene transcriptional activity in control cells and in cells treated with a plTALE$_{p300}$. This activity was demonstrated by comparing in control cells and in the treated cells the number of copies of *FXN* mRNA, the number *FXN* mRNAs normalized with GAPDH mRNAs or with the HPRT mRNA. Statistics: $p < 0.05$*, $p < 0.003$** and $p < 0.0001$****.

Figure S3

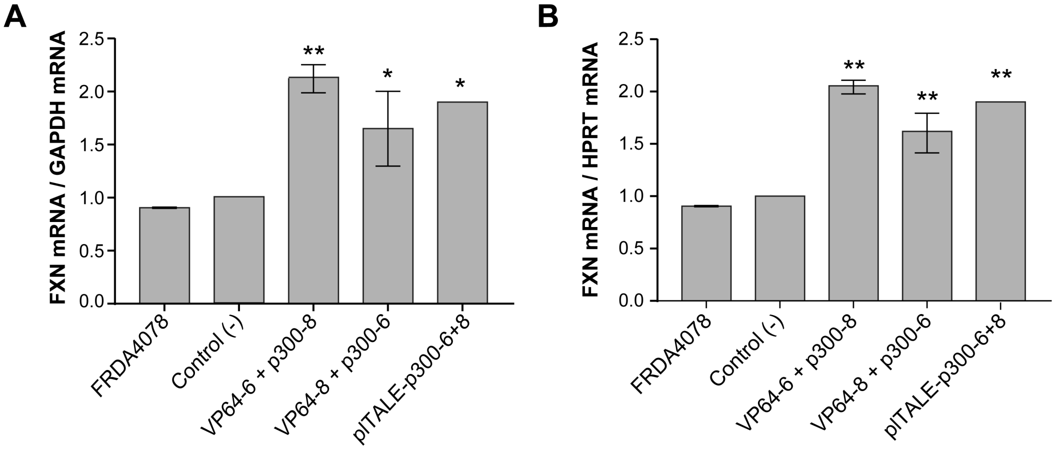

Figure S3 Absence of synergistic effects of plTALE$_{VP64}$ and plTALE$_{p300}$ in FRDA4078 fibroblasts.

A) and **B)** *FXN* gene transcriptional activity with the combined effects of plTALE$_{VP64}$ + plTALE$_{p300}$ or combined effects of 2 plTALE$_{p300s}$, which targeted sequences 6 and 8. The effectors used for this test were plTALE$_{VP64\text{-}6\text{-}15}$, plTALE$_{VP64\text{-}8\text{-}15}$, plTALE$_{p300\text{-}6\text{-}15}$ and plTALE$_{p300\text{-}8\text{-}15}$. Statistics: $p < 0.05$* and $p < 0.003$**.

Figure S4

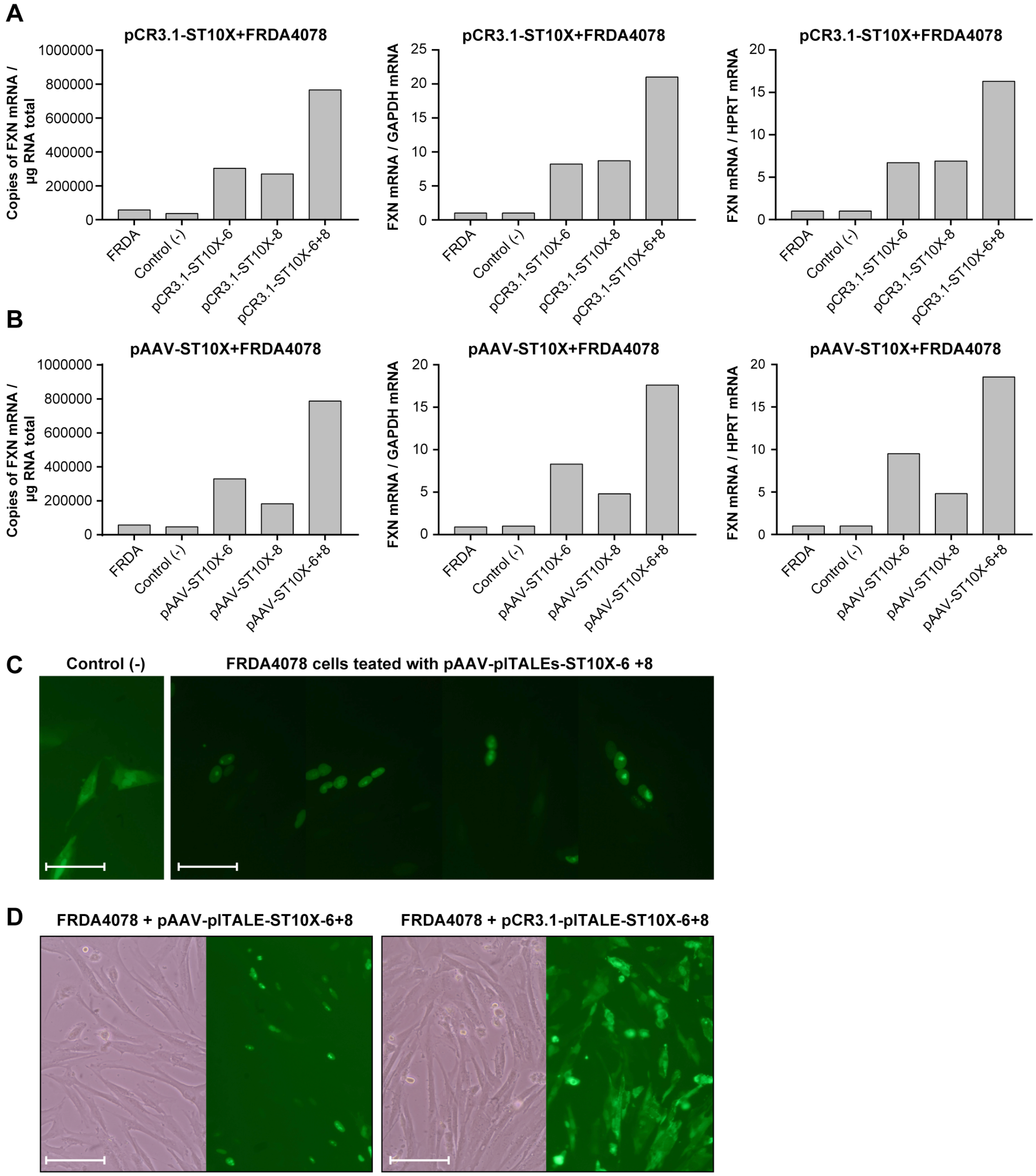

Figure S4: Induction of *FXN* gene in FRDA4078 cells with plTALE$_{STs}$ expressed by CMV or CAG promoter, and their nucleus location.

A) and **B)** *FXN* gene mRNA normalized with GAPDH or HPRT mRNA in untreated FRDA4078 (control -) or treated with 1 or 2 plTALE$_{ST10Xs}$ and scFV-sfGFP-VP64-GB1-NLS (scFv)

expressed by the pCR3.1 plasmid (promoter CMV) or the pAAV plasmid (promoter CAG). **C)** Left side: GFP fluorescence in a negative control, i.e., FRDA4078 cells treated with the pAAV-scFV-GFP plasmid, which did not express by the plTALE and with pAAV-scFv. In these cells, the GFP green fluorescence is uniform over the whole cells there no intense labelling of the nuclei. Right side: FRDA4078 cells treated with pAAV-plTALE$_{ST10X\text{-}6}$ + pAAV-plTALE$_{ST10X\text{-}8}$ + pAAV-scFv. These cells contain brighter nuclei due to plTALE$_{ST10Xs}$ targeting the specific nucleotide sequences 6 and 8 and each ST recruiting 10 scFv-sfGFP-VP64-GB1 complexes (for a total of 20 complexes). **D)** Phase contrast and GFP fluorescence of FRDA4078 cells treated with different plTALE$_{ST10X}$. Left side: FRDA4078 cells treated with pAAV-plTALE$_{ST10X\text{-}6\text{-}15}$ + pAAV-plTALE$_{ST10X\text{-}8\text{-}15}$ + pAAV-scFv, the GFP signal is localized at the plTALE targeted loci that fix 10 GFP-FV64 for each plTALE (a total of 20 GFP-VP64) leading to intense fluorescence of the nuclei. Right side: FRDA4078 cells treated with pCR3.1-plTALE$_{ST10X\text{-}6\text{-}15}$ + pCR3.1-plTALE$_{ST10X\text{-}8\text{-}15}$ + pCR3.1-scFV led to recruitment of GFP and to intense labelling of the nuclei. Scale bars in C are 50 μm and in D 100 μm.

Figure S5

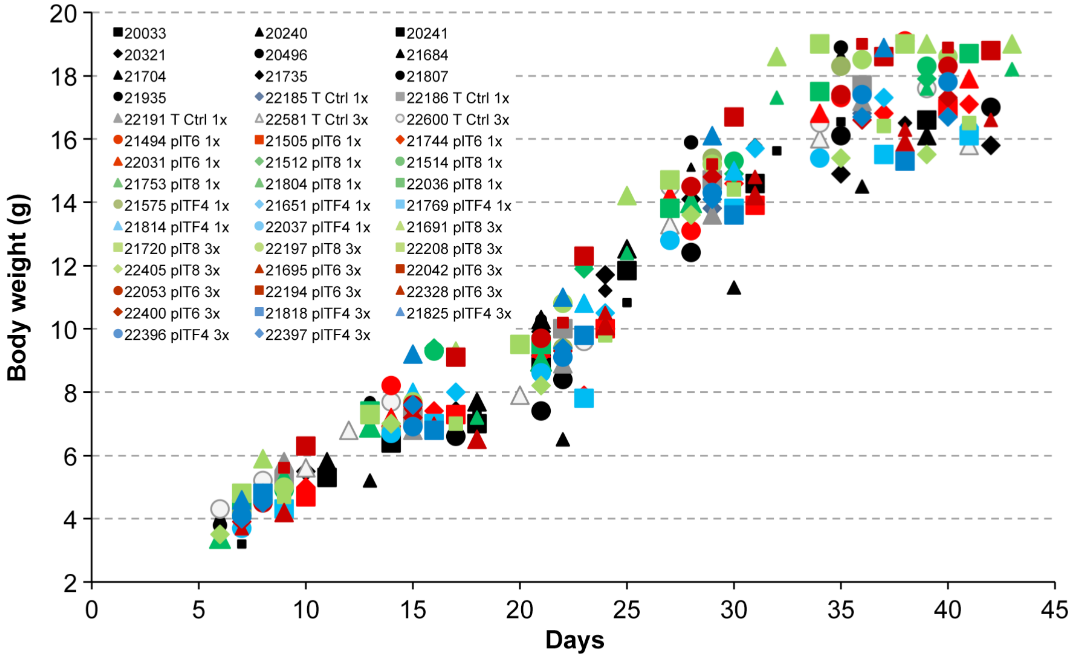

Figure S5: Body weight of untreated mice and mice treated with the different viruses.

Representative diagram of the weights of different YG8R mice treated with AAV9 that express plTALE$_{VP64-6-15}$, plTALE$_{VP64-8-15}$ or plTALE$_{VP64-F4}$ compared to control YG8R mice either untreated or treated with AAV9 (AAV9-ctrl), which did not express plTALE$_{VP64}$.

Figure S6

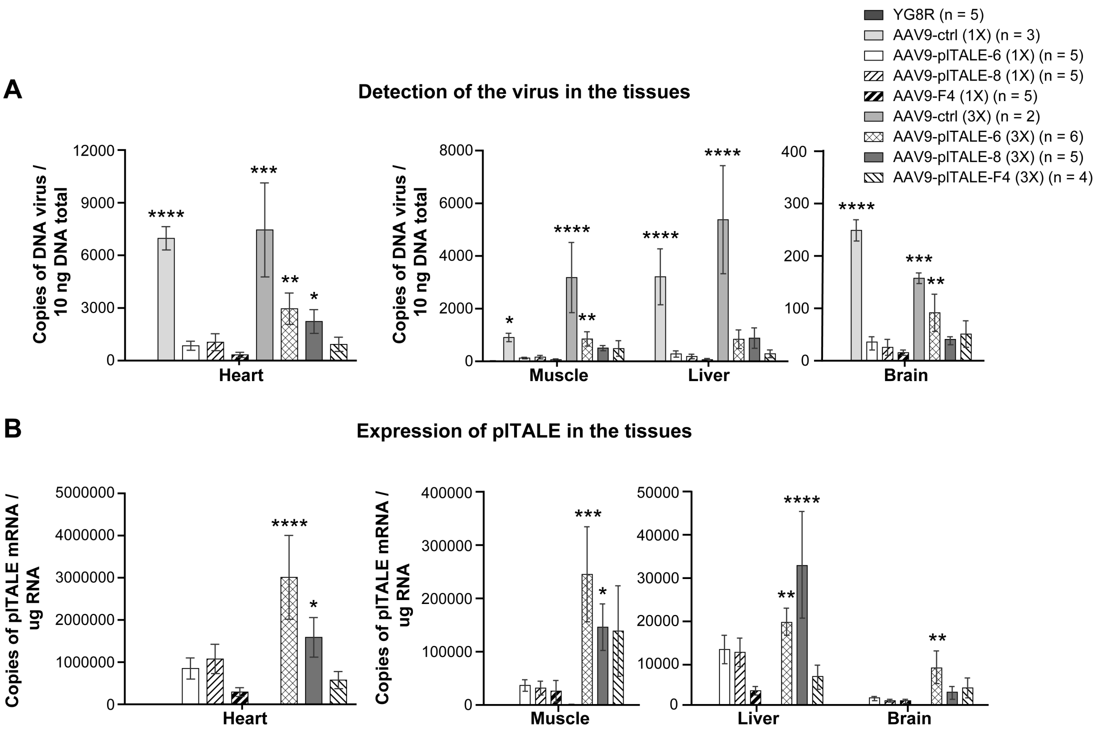

Figure S6: The distribution and expression of AAV-plTALE$_{VP64}$ *in vivo* in YG8R mice

A) Quantification of AAV9 genome in tissues: muscle, heart, liver and brain compared to untreated mice (YG8R). **B)** The expression level of plTALE$_{VP64}$ in tissues: muscle, heart, liver and brain compared to untreated mice (YG8R). Statistics: $p < 0.05$*, $p < 0.003$**, $p < 0.0003$*** and $p < 0.0001$****.

Figure S7

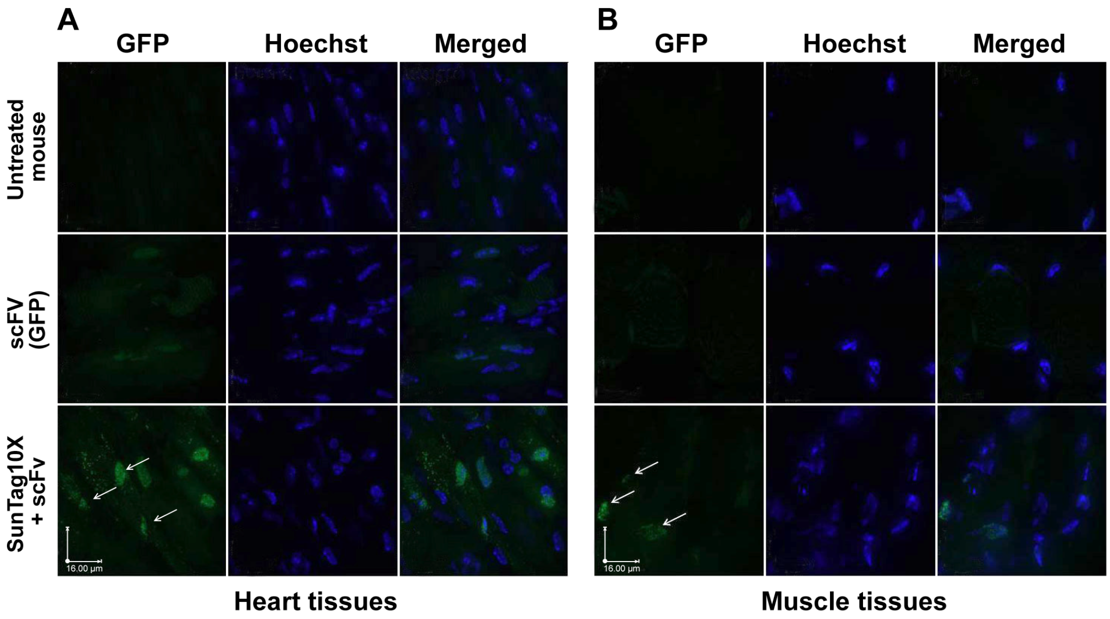

Figure S7: Labeling of the nuclei in mice treated with plTALE$_{ST10X}$.

A) and **B**) YG8R mice were injected IP with 3 different AAV viruses (i.e., 3 X 10^{11} v.p. AAV9-plTALE$_{ST10X-6}$ + 3 X 10^{11} v.p. AAV9-plTALE$_{ST10X-8}$ + 3 X 10^{11} v.p. AAV9-scFV-sfGFP-VP64). The confocal microscopy images illustrate the localization of the 20 scFV-sfGFP-VP64s recruited by plTALE$_{ST10X-6}$ and plTALE$_{ST10X-8}$ targeting the *FXN* gene promoter. The GFP fluorescence is concentrated at the nuclei (arrows) only in the heart (**A**) and the muscles (**B**) treated with AAV9s that expressed both plTALE$_{ST10X}$ and scFV-sfGFP-VP64 compared to control mouse cells where scFV-sfGFP-VP64 is expressed throughout the cytoplasm and the nucleus of the cells in the absence of plTALE$_{ST10X}$s. (Scale bar: 16 µm).

Figure S8

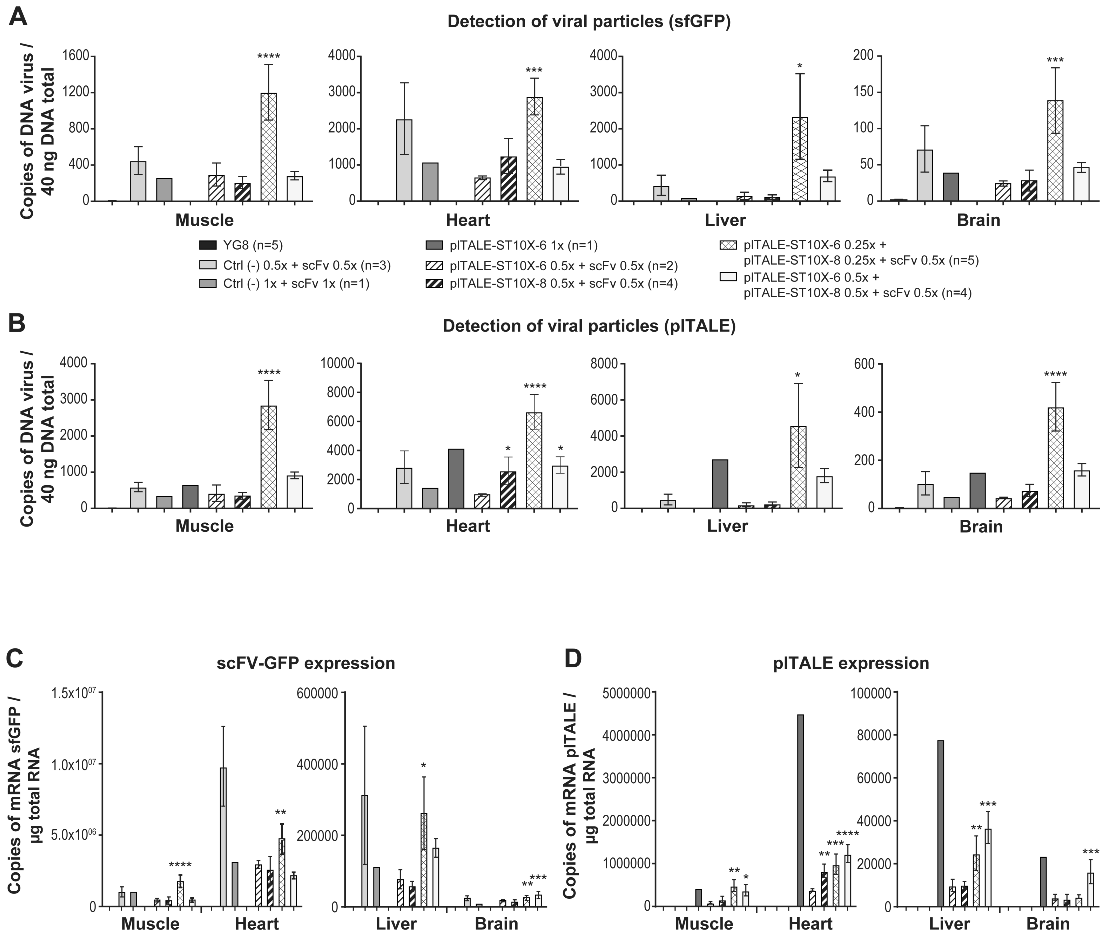

Figure S8: The distribution and expression of AAV-plTALE$_{ST10X}$ *in vivo* in YG8R mice

A) and **B)** Number of copies of AAV9 viruses, expressing either scFV or a plTALE$_{ST10X}$, in various organs of control and treated mice. **C)** and **D)** The number copies of plTALE$_{ST10X}$ mRNA and scFV-GFP mRNA in various organs of mice that received different treatments and untreated mice. Statistics: $p < 0.05$*, $p < 0.003$**, $p < 0.0003$*** and $p < 0.0001$****.

Supplementary Table 1

Gene Symbol	Description	GenBank	Size (pb)	Primer sequence 5' 3' SIAS
FXN	Homo sapiens frataxin (FXN) , nuclear gene encoding mitochondrial prote in, common region for the 3 transcrpits	NM 000144	106	AAGCCATACACGTTTGAGGACTA/TTGGCGTCTGCTTGTTGATCA
TALES			136	GAGTCGGTGTCACAGAACTCGAG/CGCCTGGTCAGGTGTCTGAGTTG
GFP			157	CCTCGTGACCACCCTGACCTAC/CTCGGCGCGGGTCTTGTAGTT
sfGFP			153	ACGCGTGCTGAAGTCAAGTTTG/CTTTTGTTTGTCTGCCGTGATGTATAC
HPRT1	Mus musculus hypoxanthine guanine phosphoribosyl transferase 1 (Hprt1)	NM 013556	106	CAGGACTGAAAGACTTGCTCGAGAT/CAGCAGGTCAGCAAAGAACTTATAGC
GAPDH	Mus musculus glyceralde-hyde -3-phosphate dehy-drogenase	NM_008084	194	GGCTGCCCAGAACATCATCCCT/ATGCCTGCTTCACCACCTTCTTG
ADNg		NT_039239	209	CACCCCTTAAGAGACCCATGTT/CCCTGCAGAGACCTTAGAAAAC
